# Novel insights into carbohydrate utilisation, antimicrobial resistance, and sporulation potential in *Roseburia intestinalis* isolates across diverse geographical locations

**DOI:** 10.1080/19490976.2025.2473516

**Published:** 2025-03-16

**Authors:** Indrani Mukhopadhya, Jennifer C. Martin, Sophie Shaw, Martin Gutierrez-Torrejon, Nikoleta Boteva, Aileen J. McKinley, Silvia W. Gratz, Karen P. Scott

**Affiliations:** a Gut Microbiology Group, Rowett Institute, University of Aberdeen, Aberdeen, UK; b Microbiology and Immunity, Institute of Medical Sciences, University of Aberdeen, Aberdeen, UK; c Centre for Genome Enabled Biology and Medicine, University of Aberdeen, Aberdeen, UK; d All Wales Medical Genomics Service, Institute of Medical Genetics, University Hospital of Wales, Heath Park, Cardiff, UK; e Department of Surgery, Aberdeen Royal Infirmary Foresterhill, Aberdeen, UK

**Keywords:** *Roseburia intestinalis*, pangenome, butyrate, carbohydrate utilization, CAZyme, antimicrobial resistance, sporulation

## Abstract

*Roseburia intestinalis* is one of the most abundant and important butyrate-producing human gut anaerobic bacteria that plays an important role in maintaining health and is a potential next-generation probiotic. We investigated the pangenome of 16 distinct strains, isolated over several decades, identifying local and time-specific adaptations. More than 50% of the genes in each individual strain were assigned to the core genome, and 77% of the cloud genes were unique to individual strains, revealing the high level of genome conservation. Co-carriage of the same enzymes involved in carbohydrate binding and degradation in all strains highlighted major pathways in carbohydrate utilization and reveal the importance of xylan, starch and mannose as key growth substrates. A single strain had adapted to use rhamnose as a sole growth substrate, the first time this has been reported. The ubiquitous presence of motility and sporulation gene clusters demonstrates the importance of these phenotypes for gut survival and acquisition of this bacterium. More than half the strains contained functional, potentially transferable, tetracycline resistance genes. This study advances our understanding of the importance of *R. intestinalis* within the gut ecosystem by elucidating conserved metabolic characteristics among different strains, isolated from different locations. This information will help to devise dietary strategies to increase the abundance of this species providing health benefits.

## Introduction

The gastrointestinal lumen is populated with heterologous species of bacteria, which have diverse functions that determine health and disease in the human host.^1^ In the colon, the vast majority of these bacteria belong to the Bacillota (previously Firmicutes) and Bacteroidetes phyla.^2,3^ One of the key functions of these bacteria is to convert complex carbohydrates that cannot be digested by host enzymes into bacterial metabolites, including the short chain fatty acids (SCFAs) acetate, butyrate, propionate and succinate.^4,5^ These SCFAs have a profound effect on ‘colonic health’ as they not only constitute one of the major energy sources for colonocytes but also exhibit distinct anti-inflammatory and anti-carcinogenic effects.^6–8^ They also play a critical role in the maintenance of barrier function in the colon.^9^ Butyrate, in particular, has an immunomodulatory function and is a known histone deacetylase inhibitor, which may explain its protective role in inflammation and carcinogenesis, respectively.^10^

Most of the butyrate producing bacteria are Bacillota with *Roseburia* spp., *Agathobacter rectalis* (previously *Eubacterium rectale*), *Anaerobutyricum hallii* (previously *Eubacterium hallii*), *Anaerostipes* spp. and *Faecalibacterium* spp. being the prominent species.^11,12^ Of these, *Roseburia* spp. and *A. rectalis* are believed to comprise nearly 24% of all bacteria in the healthy human colon.^5,13^ The importance of these critical bacteria has been highlighted in patients with inflammatory bowel disease where they are typically reduced in the mucosa and feces of affected patients.^14^ Conversely, restitution of these two species parallels clinical remission in patients with ulcerative colitis who were treated with fecal microbiota transplantation, underlining their true anti-inflammatory potential.^15^ On account of the prodigious production of butyrate by *Roseburia* spp. and *A. rectalis*, they are prime candidates as probiotic strains to revert a dysbiotic gut ecosystem.^16^

*Roseburia* spp. have been documented to have numerous health benefits in conditions ranging from inflammatory bowel diseases, colorectal cancer and atherosclerosis.^17–20^ *Roseburia intestinalis* was first identified by our group in human fecal samples as a novel saccharolytic and butyrate producing species.^21^ *R. intestinalis* has since been found to ameliorate intestinal inflammation by its selective effect on T regulatory (Treg) cells and upregulating expression of anti-inflammatory cytokines like transforming growth factor‑β (TGF‑β) and down-regulating pro-inflammatory cytokines like thymic stromal lymphopoietin (TSLP) and interleukin 17 (IL-17).^22,23^ *R. intestinalis* has also ameliorated inflammatory pathways in mice with experimental colitis and reversed 27-Hydroxycholesterol-induced learning and memory impairment in mice.^24,25^ This bacterial species is depleted in patients with Crohn’s disease, highlighting its importance in the maintenance of gut homeostasis in humans.^22^

Genomic assessment of *Roseburia* spp. together with *A. rectalis*, a closely related species, has shown multiple shared genotypic and phenotypic traits, including butyrate pathway genes and flagellar motility.^12,26^ Similar concordance was found in the genes encoding oligosaccharide transporters and regulatory elements in the genomes of these two bacterial species, forming distinct Gram-positive polysaccharide utilization loci (gpPULs).^27^ Comparative genomic assessment of core and pangenomes of different species within the genus *Roseburia* has uncovered significant differences in both degradative and biosynthetic capabilities of different species, especially with respect to carbohydrate utilization and co-factor synthesis for vitamin metabolism and utilization.^28^ Such subtle differences may determine the ability of a particular bacterial species to survive in selective ecological niches in the human colon and also determine its interaction with the other resident bacteria in the lumen. The sporulation capacity of *Roseburia* spp. is poorly characterized, yet this is an important survival and colonization mechanism. The prototype *R. intestinalis* strain L1–82 has been recognized for its potential probiotic properties and safety for use in humans.^29^ Our study aims to advance knowledge of this important bacterium and specifically focus on the pangenome assessment of *R. intestinalis* strains isolated from healthy human subjects from different geographical regions, corroborating genotypic findings with phenotypic analysis to establish the functional diversity. This will help elucidate species-specific biosynthetic and metabolic pathways of *R. intestinalis*, underpinning its potential health benefits and identifying potential dietary routes to enhance prevalence within an individual.

## Methods

### Bacterial isolates and growth medium

Two *Roseburia intestinalis* strains (PC335 and PC352) were newly isolated from two healthy adult volunteers using anaerobic culture methods after approval from the Ethics Committee of North of Scotland Research Ethics Service (Reference 17/NS/0112) and Rowett Human Studies Ethical Review Panel, as previously described.^30^ *R. intestinalis* strain PC335 was isolated from a fecal sample, and PC352 was isolated from a rectal biopsy sample. *Roseburia intestinalis* type strain L1–82 isolated from infant feces,^31^ strain M50/1 isolated from adult feces^32^ (both by our group at the Rowett Institute), and strain XB6B4 isolated from adult feces in the Unite de Microbiologie, INRAE, France, by Annick Bernalier-Donadille^33^ were included for comparison. These five strains were routinely maintained in complex, broad-range YCFAGSC bacteriological medium^34^ under anaerobic conditions in Hungate tubes (7.5 ml aliquots).

### Genomic DNA extraction

Bacterial DNA was isolated from fresh overnight cultures of *R. intestinalis* PC335 and PC352 using Promega Wizard® Genomic DNA Purification Kit (Promega) following the manufacturer’s recommendations. The DNA preparation was quantified using the Qubit™ DNA HS Assay Kit (Life Technologies, Thermo Fisher Scientific Inc.) and Qubit™ 3.0 Fluorometer (Life Technologies, Thermo Fisher Scientific Inc.). The integrity of the DNA was confirmed for all samples using the Agilent 2200 TapeStation system.

### Whole genome sequencing, assembly, and annotation

DNA extracted from *R. intestinalis* PC335 and PC352 was sequenced at the Centre for Genome-Enabled Biology and Medicine (CGEBM) at the University of Aberdeen. DNA samples were sequenced first on a single Illumina MiSeq v2 Micro flowcell (Illumina, San Diego, CA, USA) using the NextEra XT sequencing kit producing 2 × 150 bp paired end reads according to manufacturer’s protocol. Isolates were further sequenced on MinION RevD flowcells (FLO-MIN107) (Oxford NanoPore Technologies, Oxford, UK) for 72 h using the SQK-LSK108 sequencing kit following manufacturer’s instructions. The QC-filtered Illumina and MinION sequence data were then subjected to hybrid assembly using UNICYCLER 0.4.5^35^ assembler which combines both data types and attempts to circularize contigs. Assembly quality statistics were obtained using QUAST 3.1,^36^ and per-contig coverage by Illumina and MinION reads was calculated using QUALIMAP 2.1.1.^37^ Genome completeness was examined by searching the genomes for a set of 148 bacterial universal single-copy genes and quantifying the proportion of genes that had unique mapping locations, using BUSCO 3.0.0.^38^ All assemblies were processed through the PROKKA 1.11^39^ pipeline, predicting genes and tRNAs/tmRNAs/rRNAs and annotating gene predictions from the full Swissport and Pfam databases.

### Pangenome analysis to identify the core and accessory genomes

Sixteen *R. intestinalis* genomes were included in the analysis, with 14 genomes available from the NCBI genome database and two (PC335 and PC352) available from this current study. All available *R. intestinalis* genomes in the NCIMB genome database at the time of the study (in 2021) were considered for the pangenome analysis. The sequence of a further strain (43_16) was not included in the comparative analysis due to evidence that multiple genetic regions were missing within the published assembly, which would have affected assessment of the core genome. All NCBI genomes were downloaded directly in fast format. The list of final 16 genomes analyzed for this study is given in Table 1.

**Table 1.** List of *Roseburia intestinalis* genomes used in this study and annotation statistics.

*R. intestinalis* strain	Assembly Identifier	Contigs	Assembly Level	Genome Size (bp)	Coding Sequences	rRNA	tRNA	tmRNA
2789STDY5834960	14207_7#83	101	Scaffold	4322432	3895	2	56	1
AF31-21AC	ASM347551v1	79	Scaffold	4142092	3861	2	52	1
AF36-10AT	ASM347488v1	81	Scaffold	4303042	3911	2	63	1
AM22-21LB	ASM347104v1	68	Scaffold	4112416	3701	2	49	1
AM37-1AC	ASM346772v1	60	Scaffold	4253056	3939	3	59	1
AM43–11	ASM346703v1	91	Scaffold	4337927	3996	3	56	1
BIOML-A1	ASM971834v1	79	Contig	4348274	3977	2	60	1
BSD2780061689_150309_G12	ASM1555478v1	157	Scaffold	4171472	3726	2	56	1
J1101437_171009_C5	ASM1555578v1	95	Scaffold	4570840	4235	2	57	1
L1–82	*Roseburia intestinalis* strain L1–82	1	Complete	4493348	4067	15	69	1
M50/1	ASM20999v1	1	Chromosome	4143550	3794	2	51	1
MSK.17.84	ASM1330044v1	161	Contig	4374175	4063	2	63	1
PC335	This study	11	Contig	4411266	4042	14	77	1
PC352	This study	16	Contig	4422834	4134	13	59	1
SNUG30017	ASM975952v1	79	Contig	3902409	3518	4	72	1
XB6B4	ASM21065v1	1	Chromosome	4286292	3881	2	62	1

To ensure standardization across assemblies, all genome assemblies were re-annotated from scratch using Prokka (version 1.13.7)^39^ with the most recent Pfam database^40^ provided. Pangenome analysis was then carried out using Roary (version 3.12.0)^41^ classifying genes as core, soft-core, shell, and cloud genes according to their varying presence among the genomes analyzed. Thus, genes present in >99% strains (all 16 strains) were designated as core genes; genes present in 95% to 99% (15) strains were classified as soft-core genes; shell genes were present in 15% to 95% (3–15) strains, and genes present in less than 15% of the strains analyzed (less than 2 genomes) were assigned as cloud genes. The core genome alignment produced by Roary was used for phylogenetic analysis of the relatedness of the strains using RAxML (version 8.2.12)^42^ using the model GTRGAMMA and 100 bootstraps. A circular map of the 16 *R. intestinalis* genomes was constructed using the Proksee web server (https://proksee.ca/).^43^

Carbohydrate active enzymes (CAZymes) were identified in the pangenome using dbCAN2^44^ using search tools HMMER (e-value < 1e-15, coverage > 0.35), DIAMOND (e-value < 1e-102), Hotpep (Frequency > 2.6, Hits > 6) and CGCFinder (Distance ≤ 2, signature genes = CAZyme + TC). These parameters are based on established benchmarks enabling accurate functional annotation. All genes with a consistent positive identification by two or more tools were used for further analysis. Predictions of gpPULs were achieved by further analyses of genes indicated using >2 tools to be GH2, GH3, GH13, GH23, GH43, or GH78. Up to 15 genes up and downstream of each predicted GH were identified, or up to the limits of the contig, and the PROKKA gene annotations were tabulated. gpPULs containing specific glycoside hydrolase genes alongside genes encoding carbohydrate transport enzymes and regulatory genes were then manually curated.

To identify potential antimicrobial resistance genes (AMR) across the pangenome, the consensus sequence of all 11,814 genes were first converted to protein sequences using transeq (part of EMBOSS package; version 6.6.0),^45^ and AMR genes were identified by comparison to the Comprehensive Antibiotic Resistance Database (CARD) with RGI (version 5.1.0),^46^ using embedded detection thresholds. Further, to identify potential mobile genetic elements (MGEs) across the pangenome, the translated protein sequences of all genes were compared to the ISFinder database^47^ using BLASTP (version 2.9.0)^48^ with an e-value cut-off of 1e-05 and a percent identity cut-off of 70%. Custom Python scripts were then used to identify the top hit per gene based on the smallest e-value (with the highest percentage identity used when e-values were identical).

### Functional annotation of genomes

The consensus gene sequence for all of the *R. intestinalis* genes in the core, shell and cloud gene sets were functionally categorized by comparison to the Clusters of Orthologous Groups (COG) database (version COG2020)^49^ using BLASTX (version 2.9.0)^48^ with an e-value cutoff of 1e-06. Custom python scripts were used to filter all hits with a percentage identity >70% and to then select the “top hit” for each gene based on the smallest e-value (when e-values were equal, highest percentage identity was used to determine top hit). These top hits were then matched to the COG identifier and functional group using custom python scripts.

### Functional annotation, homology and synteny analysis of tet(O) regions

Functional annotation of genes surrounding *tet*(O) in all strains that contained it was performed with BLASTx with standard settings, and functional identification was accepted with >95% cover and sequence homology as cutoffs. Homology and synteny of the *tet*(O) containing regions was performed by aligning each region to its closest relative with BLASTn with standard settings and visualizing the resulting hit table (text) with the Artemis Comparison Tool.^50^

### Growth assays on different carbohydrate substrates

The five *R. intestinalis* strains that were available within the Rowett (PC335, PC352, XB6B4, M50/1 and L1–82) were tested to confirm specific phenotypes. Sterile, polystyrene, flat-bottom, 96-well tissue culture plates (Costar) were used. Growth kinetics in the 96-well microplates was monitored (as turbidity) with a computer-controlled plate reader (BioTek EPOCH 2) running Gen5 Microplate data collection and analysis software (version 3.10) and a 650-nm filter. Growth of the seed cultures was monitored on a Dynamica Halo-Vis 20 spectrophotometer by measuring the optical density at 650 nm (OD_650_) of 7.5 ml samples.

### Short-chain and branched chain fatty acid (SCFA and BCFA) quantification

SCFA/BCFA formation was assessed in culture supernatants by gas chromatography (GC) following standard procedures.^51^ Bacterial cultures were grown in triplicate on YCFAGSC overnight and OD_650_ readings were taken to confirm equivalent growth. OD_650_ readings ranged from 2.12 to 2.46, with a mean of 2.26 and a median of 2.23. The SCFA/BCFA concentrations (mM) were calculated in duplicate from standard curves based on peak size and retention time. Concentrations measured in a media-only control are subtracted from concentrations measured in individual bacterial cultures to give a net concentration that is a balance of production and consumption. An internal standard of 2-ethyl butyric acid and an external standard (a mixture of volatile fatty acids and salts [SCFA: 30 mm Acetic acid, 20 mm Propionic acid, 20 mm Butyric acid, 5 mm Valeric acid; BCFA: 5 mm iso-Valeric acid, 5 mm Iso-Butyric acid] plus salt solutions of 10 mm sodium formate; 10 mm Lithium lactate and 10 mm sodium succinate) was included in each GC run.

### Antibiotic susceptibility

Antibiotic susceptibility testing was conducted using fresh overnight bacterial cultures grown in YCFAGSC medium. Isolates were screened for possible resistance against a selection of 11 antibiotics as per EFSA guidelines^52^ using a slightly modified version of the agar disc diffusion method.^53^ The turbidity of each growing bacterial suspension was adjusted by dilution with sterile media to match the turbidity standard of 0.5 McFarland units. Petri plates (9 cm diameter), containing 20 ml of YCFAGSC agar (2%), were overlaid with 10 ml of M2GSC soft agar (1%) containing 50 µl of the diluted bacterial culture. Antibiotic discs (Oxoid) were placed on the inoculated plates using the Oxoid disc dispenser. Following 48 h anaerobic incubation at 37°C, inhibition zones around the discs were measured. The tests were conducted and results interpreted according to EUCAST guidelines.^54^

Tests to ascertain the minimum inhibitory concentrations of tetracycline and erythromycin were performed in the same way, but antibiotic-containing discs were replaced with ETEST strips (BioMérieux) impregnated with 0.016–256 µg/ml tetracycline or erythromycin.

### Detection of spores using transmission electron microscopy (TEM)

*R. intestinalis* strains (L1–82, M50/1, PC335 and PC352) were inoculated into fresh YCFAGSC liquid medium from frozen stocks and grown for 72 h. These cultures were subjected to both heat-shock (80°C, 20 min) and oxygen shock by subsequent exposure to air (30 min). Cultures were then pelleted by centrifugation and treated with primary and secondary fixatives following previously published methods.^55^ Sporulation images were documented using a JEOL 1400 plus; TEM and AMT UltraVUE camera.

### Hanging drop microscopy

Motility of the bacterial strains was detected using hanging drop microscopy as described previously.^56^ Briefly, a small drop of bacterial suspension was placed onto a sterile cover slip. The cover slip was then carefully inverted over the well of a concave microscope slide, creating a sealed, humid chamber. This setup allowed observation of bacterial movement in a free-floating state under a light microscope using oil immersion.

## Results

### Roseburia intestinalis pangenome analysis

**Figure 1.**
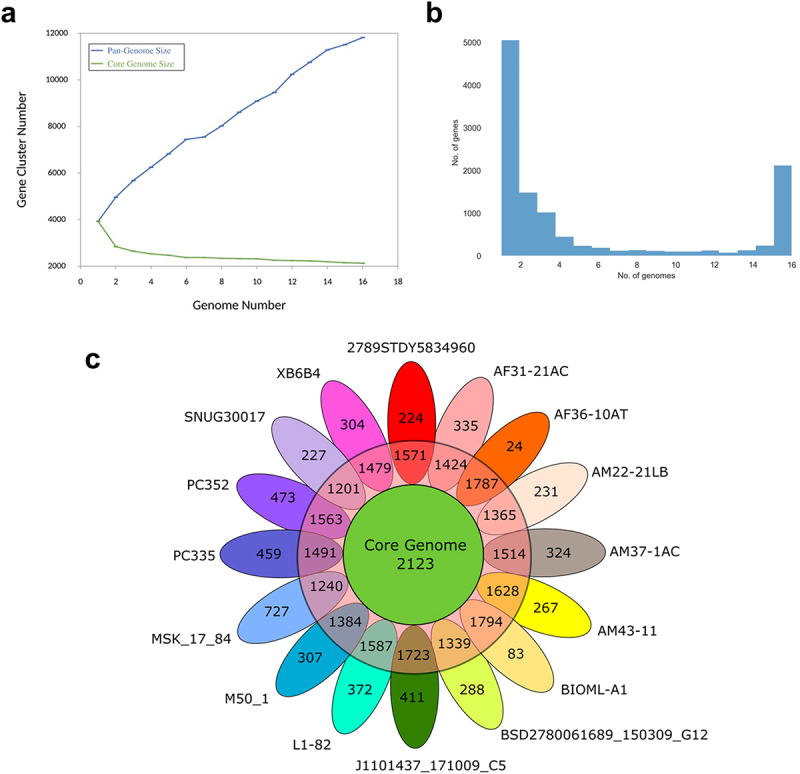
Pangenome analyses of 16 *Roseburia intestinalis* strains. a) pangenome accumulation curves. The blue line denotes the number of unique genes discovered with the sequential addition of new genomes. The green line denotes the number of core genes discovered with the sequential addition of new genomes. b) gene frequency plot of *R. intestinalis* genomes. c) genomic diversity of 16 *R. intestinalis* strains shown in a flower plot. Each strain is shown as a petal. The number of core genes (present in >99% strains) is shown in the center (green circle). Overlapping regions in the red circle show the shell genes (present in 15% to 95% of strains) conserved only within several strains. The numbers in non-overlapping portions show the number of unique strain-specific genes. The strain name is located beside each petal.

Pangenome analysis to determine the over-lapping and strain-specific genes of the 16 selected genomes of *R. intestinalis* identified 11,814 unique protein-coding genes across all genomes. The number of coding sequences in an individual genome ranged from 3518 to 4235 (Table 1). The gene accumulation curve showed that the number of genes assigned to the core genome decreased progressively with the addition of new strains, while the total pangenome increased (Figure 1(a)). The change in both curves slowed down because the pan-genome of *R. intestinalis* was in an open state, indicating that more unique genes would be added along with the addition of more new strains. The gene frequency plot depicting the distribution of genes across the strains is shown in Figure 1(b). The pangenome analysis revealed that a core genome containing 2,123 genes was present in all 16 *R. intestinalis* strains and in addition, 3,134 shell genes and 6,547 cloud genes were identified (Figure 1(c)). Interestingly, 5,056 of the cloud genes were unique to one genome among these strains (Table 2). No soft-core genes were identified, and the core and shell genes accounted for 84% of the pangenome, indicating that the genomes of the strains were remarkably conserved. The notable differences in the number of cloud genes between strains, ranging from only 24 genes in AF36-10AT to 727 genes in MSK.17.84 (Figure 1(c)), suggests that these two strains may have adapted to different habitats and that strain-specific genes may have been acquired through horizontal gene transfer from other species. Exclusively absent genes, classified as genes present in all other genomes, but not the genome specified were also identified in all but one strain (Table 2).

**Table 2.** Pangenome summary statistics per *R. intestinalis* strain.

*R. intestinalis* strain	No. of core genes	No. of shell genes	No. of cloud genes	No. of unique genes	No. of exclusively absent genes
2789STDY5834960	2123	1303	492	224	9
AF31-21AC	2123	1275	484	335	14
AF36-10AT	2123	1521	290	24	0
AM22-21LB	2123	1217	379	231	24
AM37-1AC	2123	1340	498	324	17
AM43–11	2123	1440	455	267	14
BIOML-A1	2123	1511	366	83	5
BSD2780061689_150309_G12	2123	1231	396	288	7
J1101437_171009_C5	2123	1535	599	411	5
L1–82	2123	1369	590	372	5
M50_1	2123	1267	424	307	39
MSK_17_84	2123	1098	869	727	8
PC335	2123	1293	657	459	10
PC352	2123	1340	696	473	30
SNUG30017	2123	1045	383	227	36
XB6B4	2123	1326	457	304	26

### Phylogeny and genome comparisons

**Figure 2.**
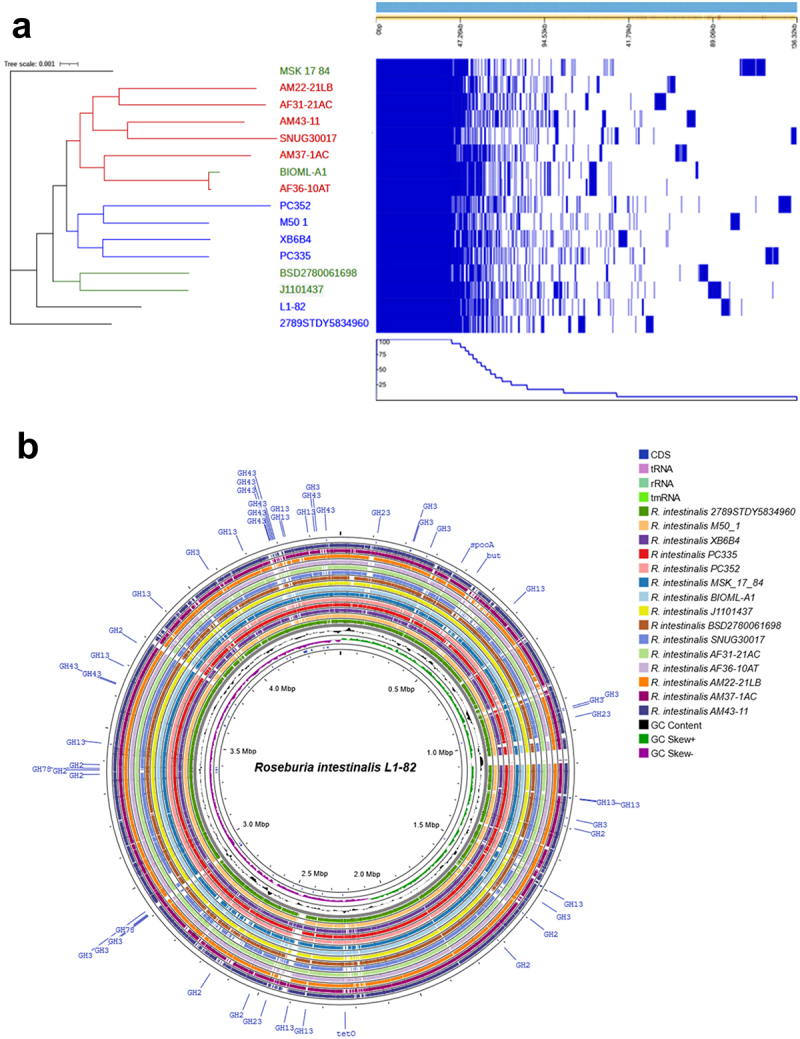
Genomic relatedness of *Roseburia intestinalis* strains. a) Phylogenetic tree visualized alongside the pangenome gene presence/absence results. The strains originating from Asia are shown in red, the ones from Europe are in blue and the ones from America are in green. b) circular map showing a comparison of the *Roseburia intestinalis* genomes.

A phylogenetic tree based on the core genome identified up to three distinct clades potentially coinciding with three different geographical locations where the strains were isolated. The first clade consisted of six strains isolated from Asia (AM22-21LB, AM37-1AC, AF36-10AT, SNUG30017, AM43–11 and AF31-21AC), and the second clade consisted of four strains from Europe (M50/1, XB6B4, PC352 and PC335). A possible third clade comprised two strains isolated from America (J1101437_171009_C5 and BSD2780061689_150309_G12) (Figure 2(a)), while a further three strains were not part of any clade. This provides the first evidence of potential geographic stratification of *R. intestinalis* strains on a global scale, with strains isolated in Europe and Asia clustering separately. Detailed comparison of the sixteen *R. intestinalis* genome sequences is illustrated in the form of a circular map in Figure 2(b).

### Functional traits of the R. intestinalis core and accessory genes

The proportion of genes assigned to specific functional groups differed across the core, shell and cloud genomes (Supplementary Table S1; Supplementary Figure S1). Functional enrichment using COG showed that the most conserved predicted proteins were those involved in primary metabolism and DNA processing functions. Notably, “translation, ribosomal structure and biogenesis” formed the largest portion of core genes (13.38%), with the proportion of this category of genes reduced in the shell (4.63%) and cloud (3.13%) genomes. Likewise, the proportion of genes with the function “energy production and conversion” also decreased from core to shell and cloud genomes. The proportion of “defense mechanisms” genes increased from core, to shell, to cloud genome, and this pattern was also observed for genes involved in “transcription”, “replication, recombination and repair”, and “cell wall/membrane/envelope biogenesis”. Interestingly, none of the genes associated with the “mobilome: prophages, transposons” was found in the core genome, with increasing proportions detected in the shell (4.92%) and cloud (12.53%) genomes.

There were clear differences in the proportion of unique genes within different functional categories across each genome (Supplementary Table S2; Supplementary Figure S2). For instance, genes related to “transcription” formed a relatively high proportion of the unique genes in most genomes, but <10% in AF36-10AT, BIOML-A1, and J1101437_171009_C5. The functional category of “exclusively absent” genes varied between each genome (Supplementary Table S3; Supplementary Figure S3).

### Carbohydrate utilisation by R. intestinalis

**Figure 3.**
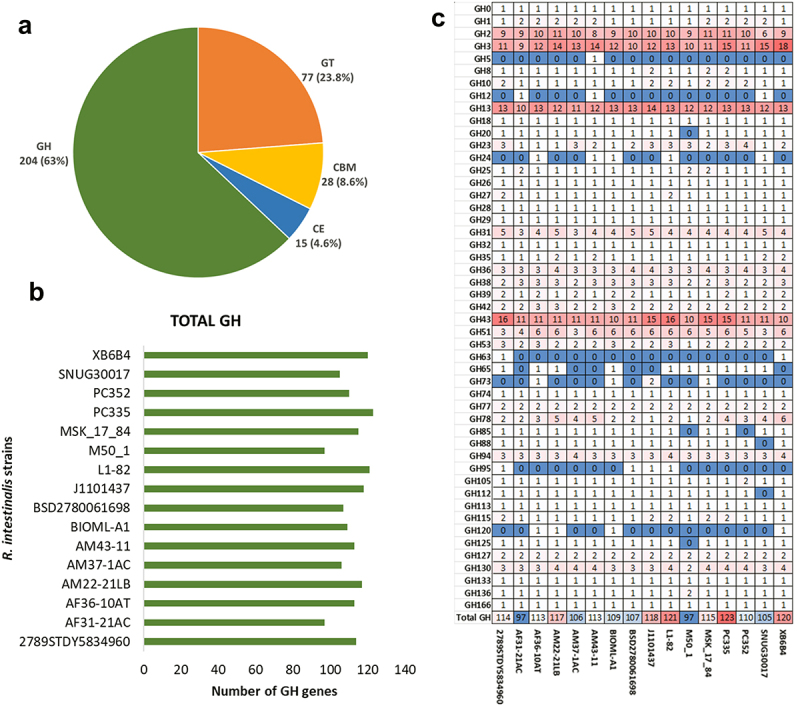
Carbohydrate active enzymes (CAZymes) a) percentage of each category of CAZyme (CBM – carbohydrate-binding module, CE – carbohydrate esterase, GH – glycoside hydrolase, GT – glycosyl transferase) b) total GH genes identified in each *R. intestinalis* genome c) number of GH genes across genomes with red indicating the most and blue indicating the least.

Previous studies have reported that *R. intestinalis* can ferment dietary fibers to produce butyrate under anaerobic conditions, specializing in utilizing selective glycans like xylan and β-mannan.^27,57^ However, most of these studies have been carried out using the type-strain L1–82 and there is limited information available on the carbohydrate utilization pattern of other *R. intestinalis* strains. In the present study, *R. intestinalis* genes were systematically identified against the CAZy database to obtain a comprehensive understanding of the carbohydrate degradative ability. A total of 295 genes within the pangenome contained at least one CAZy domain with 23 genes containing two or more domains. Of these domains, 63.0% were glycoside hydrolases (GH), 23.8% were glycosyl transferases (GT), 8.6% were carbohydrate-binding modules (CBM) and 4.6% were carbohydrate esterases (CE) (Figure 3(a)). No polysaccharide lyases (PL) were identified in the *R. intestinalis* pangenome. Ninety-six CAZy domains were present in all strains, indicating considerable inter-strain conservation.

Each individual genome of the 16 human *R. intestinalis* strains encoded between 97 and 123 glycoside hydrolases (GHs) (Figure 3(b), Supplementary Figure S4). Most of these GH domains (over 50%) represented just six CAZyme families; GH3 (12.75%), GH43 (11.76%), GH2 (9.8%), GH13 (6.37%), GH23 (5.39%) and GH78 (5.39%). Among these families, GH3, GH43 (and additionally GH10 and GH51) are mainly involved in xylan utilization; GH13 enzymes with starch utilization; GH2 (and additionally GH31 and GH130) in mannose utilization and GH78 with rhamnose utilization. The presence of multiple copies of these specific gene families (GH2, GH3, GH13, GH43) in all isolates indicates that starch, xylan and mannose represent core substrates for *R. intestinalis* growth. Of the total 204 GH genes identified, 65 were from the core gene set across all 16 genomes, with a further 33 genes present in 10 or more genomes (Figure 3(c)).

The first step in glycan degradation often requires attachment of glycans to the bacterial cell surface and is typically mediated by carbohydrate-binding modules (CBMs). The most common CBMs identified in the *R. intestinalis* pangenome were associated with glycogen binding (CBM48), xylan binding (CBM6, CBM22, CBM86), starch binding (CBM34 and CBM83), mannan binding (CBM23 and CBM27) and L-rhamnose binding activity (CBM67). Of the CBMs identified, only two (both CBM50, associated with chitin or peptidoglycan degradation) were present as single-domain genes in the shell genome of 14 and 3 strains. All strains harbored single genes containing CBM50, except isolate SNUG30017 where two separate genes contained a CBM50 domain. The remaining 26 CBMs were part of multi-domain genes. In fact, 20/23 dual-domain genes contained a CBM linked to a GH. Two genes each contained four domains: CBM86, CBM22, GH10 and CBM9 that are all associated with xylan utilization. Although these genes were in the shell genome (identified in 12 and 5 isolates), all 16 genomes possessed a version of this multi-domain gene, with only one genome encoding both genes (PC352). Five multi-domain genes were part of the core genome, present in all 16 isolates. Four of these were involved in starch utilization, combining GH13 with CBM43 (and one with CBM48) while the fifth containing CBM27, CBM23 and GH26 is likely to be involved in mannan utilization. The rhamnose utilization domains CBM67 and GH78 were always detected together comprising part of three cloud genes, one found in three isolates (AM37, L1–82 and XB6B4) and one in each of the single strains PC335 and PC352. The final three multi-domain genes each contained two GT domains. Two of these contained GT8 and GT111 (a β-1,3-galactofuranosyltransferase), while the other one containing GT2 and GT4 is likely to have multiple activities.

**Figure 4.**
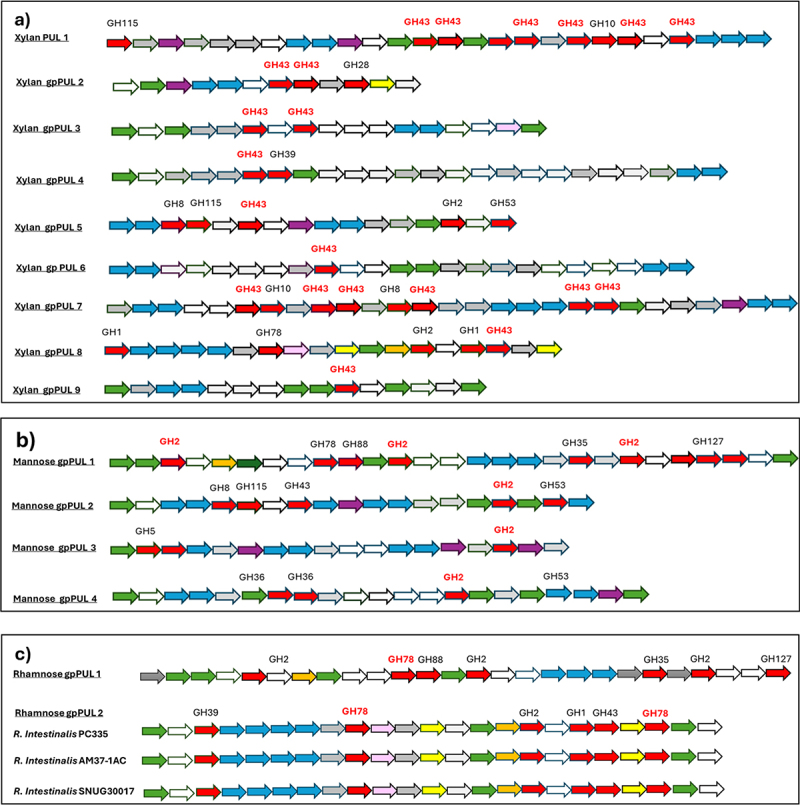
*Roseburia intestinalis* predicted gpPULs. Schematic representation of gpPULs concerned with a) xylan, b) mannose and c) rhamnose utilization. Glycoside hydrolase (GH) genes are colored red, and those associated with utilization of the key substrates further labeled in bold red text. ABC-transporter system component genes are colored blue. Transcriptional regulator genes are colored green. Xylose/rhamnose isomerase genes are colored yellow. Orange color represents the MFS/sugar transport genes, pink color represents the Methyl-accepting chemotaxis protein (MCP) signaling domain protein, and purple are bacterial extracellular solute binding genes. Hypothetical genes are colored gray. Genes with other unrelated functions are colored white.

In addition to multi-domain genes, *Roseburia* species have been shown to contain loci containing catalytic enzymes alongside genes encoding regulatory and transport functions, known as gpPULs.^27^ The 16 *R. intestinalis* genomes contained multiple potential gpPULs (Supplementary file 1), some of which contained the highly conserved CAZyme families. Specific gpPULs involved in xylan, mannose and starch utilization were identified, many present in all 16 strains (Figure 4, Supplementary Figure S5). Xylan utilization is highly conserved in *R. intestinalis* and nine specific xylan gpPULs were identified, frequently containing multiple GH43 CAZymes. Xylan gpPUL 1 and gpPUL 7 both contained 6 different GH43 enzymes but were each only present in single strains (L1–82 and PC335 respectively). In contrast, xylan gpPULs 2, 3, 4, 5 and 6 were present (and highly conserved) in most strains (Figure 4(a)). Mannose gpPUL 1, identified in all isolates, contained three GH2 domain enzymes, and an additional six CAZymes. Mannose gpPUL 2 was also found in all 16 isolates, while mannose gpPULs 3 and 4 were present in 7 and 10 isolates, respectively (Figure 4(b)). Starch gpPULs 1, 2 and 5 were also found in all strains, while starch gpPULs 3 and 4 were identified in 8 and 12 strains, respectively (Supplementary Figure S5). Only two gpPULs potentially involved in rhamnose utilization were identified. One of these (rhamnose gpPUL 1) was found in all strains and corresponds to mannose gpPUL 1. In contrast, rhamnose gpPUL 2, which contains two GH78 enzymes and a further four CAZymes, was only found in three strains (Figure 4(c)). Some of these gpPULs also contained chemotaxis proteins that may facilitate utilization of the specific substrates in the competitive gut environment.

### Growth assays to confirm functionality of CAZy genes identified

**Figure 5.**
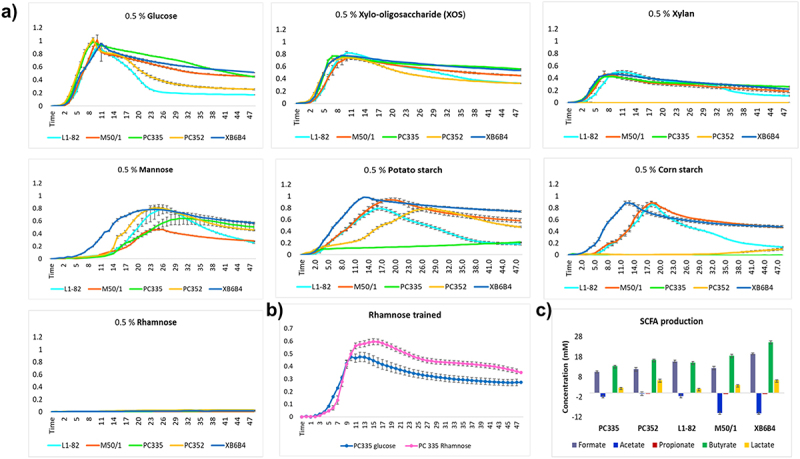
Growth kinetics of five *R. intestinalis* strains on different carbohydrate substrates (0.5%). b) growth of the ‘trained’ PC335 strain on rhamnose. c) Short Chain Fatty Acid concentrations measured in *R. intestinalis* strains cultured in YCFAGSC media. Net concentrations shown are relative to a media-only control and are a balance of production and consumption.

In order to ensure that the most abundant GH and CBM enzymes identified from the CAZyme analysis were functional, we tested the growth of the five available *R. intestinalis* strains (L1–82, M50/1, XB6B4, PC335 and PC352) on YCFA media supplemented with single different carbohydrate substrates (Figure 5(a)). All five strains grew very well on the control substrate 0.5% glucose (average maximum OD_650_ 1.0). They were also all able to grow well on 0.5% xylo-oligosaccharide (XOS) (avg. max. OD_650_ 0.8) and to a moderate level on 0.5% Xylan (avg. max. OD_650_ 0.5). Strain-specific differences were noted for the growth on 0.5% mannose with XB6B4, PC352 and L1–82 strains showing the highest growth (avg. max. OD_650_ 0.78), whereas moderate growth was noted for PC335 strain (avg. max. OD_650_ 0.67) and lower growth for M50/1 (avg. max. OD_650_ 0.48). Moreover, there was shorter lag phase observed for XB6B4 strain compared to the other strains. Most strains grew well on 0.5% potato starch (avg. max. OD_650_ 0.9), except PC335 did not grow in repeated tests and PC352 had a long (12–24 h) lag period. In contrast, the strains varied in ability to grow on 0.5% corn starch with the older isolates (L1–82, M50/1, XB6B4) growing very well (avg. max. OD_650_ 0.8) while the newer isolates (PC335 and PC352) did not grow at all. None of the strains were initially able to grow on 0.5% rhamnose. However, when PC335 was trained through repeated inoculations to grow on rhamnose under laboratory conditions, it became able to grow well (avg. max. OD_650_ 0.6) (Figure 5(b)). The other strains tested (L1–82, PC352, M50/1) could not be trained to grow on rhamnose. Interestingly, of these five strains, only strain PC335 contained the rhamnose gpPUL 2.

### Short chain fatty acid (SCFA) analysis

Genome analysis indicates that the Butyryl-CoA:acetate CoA-transferase gene was a single-core gene, present in the genomes of all isolates analyzed. None of the strains contained the butyrate kinase gene consistent with previous work demonstrating the butyrate synthesis pathway via the CoA-transferase gene in *Roseburia* species.^12^ Analysis of SCFA production confirmed that all strains produced butyrate, with concentrations ranging from 13.4 mm to 25.3 mm. In pure culture, strains also produced significant amounts of formate (10.5–19.4 mm) and lower amounts of lactate (2.0–6.3 mm) (Figure 5(c)). *R. intestinalis* strains M50/1 and XB6B4 also consumed significant amounts (10 mm) of acetate. None of the strains produced propionate, which was consistent with the lack of any genes linked to propionate production.

### Antibiotic resistance and mobile genetic elements in R. intestinalis

Seven genes potentially encoding antimicrobial resistance (AMR) proteins were identified in the *R. intestinalis* pangenome which included the tetracycline-resistance genes *tet*(O) and *tet*(40) and erythromycin resistance gene *ermB* (Supplementary Table S4).

All the tetracycline resistance genes were present in the shell of the pangenome indicating acquisition may have been due to environmental pressure. The *tet*(O) gene was present in 10 of the 16 *R. intestinalis* strains (L1–82, AF31-21AC, AF36-10AT, AM22-21LB, AM43–11, BSD2780061689_150309_G12, BIOML-A1, PC335, PC352 and XB6B4) while the *tet*(40) gene was present in four strains (AF31-21AC, AM22-21LB, AM43–11 and L1–82). Notably, the *tet*(40) genes were located downstream of *tet*(O), and never occurred alone. The *tet*(40) gene was predominantly detected in Asian strains (3 out of 4), with a single occurrence in a European strain L1–82 and none in the American strains. The *erm*(B) gene was found in the shell genome of only three strains (AF31-21AC, AM37-1AC and AM43–11), all Asian isolates and two of which also contained *tet*(O).

The composition of the AMR gene neighborhood can affect the transfer of AMR genes between gut bacteria. For instance, genes that encode conjugative transposons or plasmids can facilitate the transfer of AMR genes between different bacteria through horizontal gene transfer events, while insertion sequences may facilitate their movement within the genome. A total of 48 genes were identified with significant hits to the ISFinder database (Supplementary Table S5), of which 44 were identified as transposases. Thirty-eight of these genes were present in the cloud gene set of either one or two genomes and none in the core genome.

**Figure 6.**
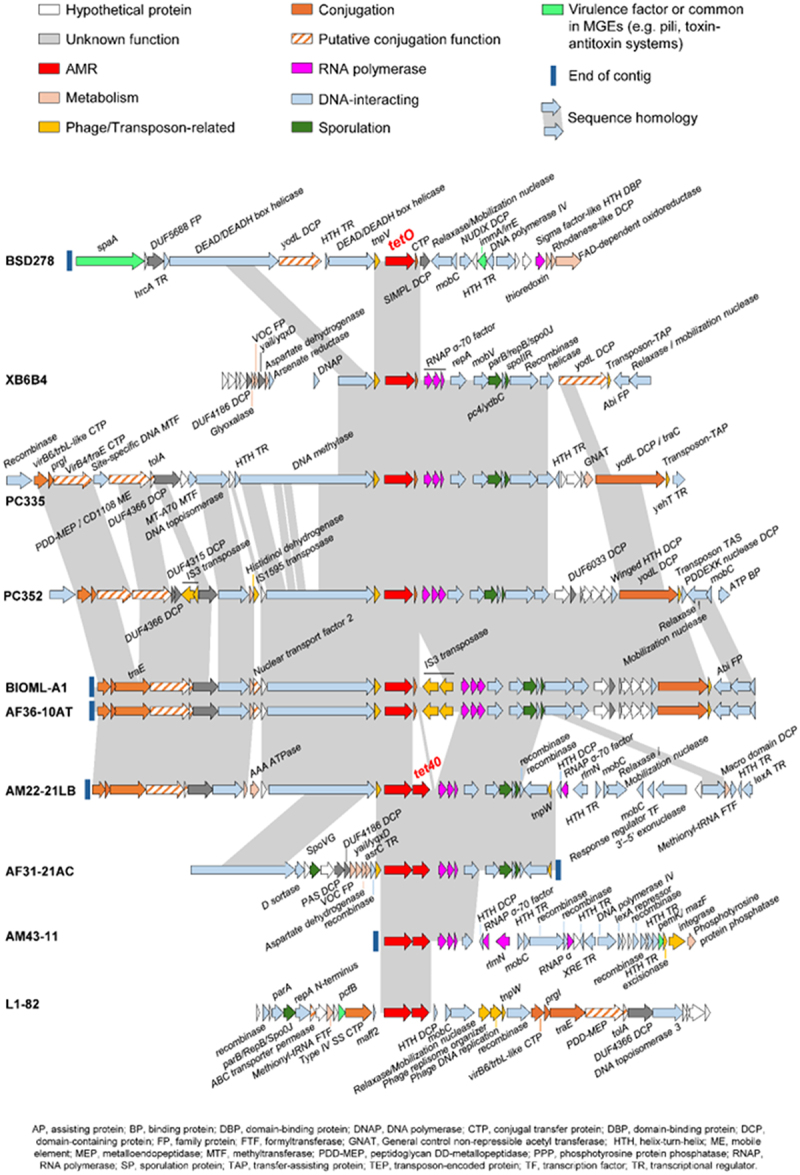
Comparison of the genetic environment of the *tet*(O) gene in *R. intestinalis* strains.

The presence of any possible mobile genetic elements (MGE) that could potentially spread tetracycline resistance from the 10 *R. intestinalis* strains was investigated by specifically analyzing the genetic neighborhood surrounding the *tet*(O) and *tet*(40) genes. The putative functions of the surrounding genes were identified through homology using BLASTp analysis of 10 open reading frames (ORFs) upstream and downstream of *tet*(O) and/or *tet*(40) genes. A schematic diagram representing these ORFs is presented in Figure 6. The transposon-encoded gene *tnpV* was located immediately upstream of the *tet*(O) gene in 8 of the 10 genomes. A gene encoding the conjugative transfer protein (CTP) was present immediately downstream of *tet*(O) genes in six of these genomes but was absent for the four strains containing the *tet(*40) gene. There was considerable conservation of the genetic neighborhood downstream of *tet*(O) (plus/minus *tet*(40)), particularly in isolates close together in the phylogenetic tree (Figure 2). Many of the genes identified have potential roles in gene mobility (helicases, relaxases and recombinases), suggesting the presence of a mobile genetic element. A Venn diagram (Supplementary Figure S6) was used to visualize the main features distinguishing the genome sequences. In strain AM43–11, the contig sequence containing the *tet*(O) and *tet*(40) genes was truncated immediately upstream of *tet*(O) so the presence of the *tnp*V gene could not be determined.

### Antibiotic resistance phenotype confirmation

The antibiotic resistance phenotypes of the five strains in our culture collection were tested to verify the AMR genotypes. Disc assays confirmed that strain M50/1 (no tetracycline resistance gene) was sensitive to tetracycline, while the remaining four strains which all contained the *tet*(O) gene were resistant to tetracycline (30 µg/ml) (Table 3). Tests to determine the minimum inhibitory concentrations (MIC) further confirmed the sensitivity of strain M50/1 that could not grow in tetracycline concentrations exceeding 0.019 μg/ml. The MIC for strains XB6B4, L1–82 and PC352 ranged from 16 to 32 μg/ml while strain PC335 was able to grow up to 128 μg/ml. All but one of the strains tested were sensitive to erythromycin (15 µg/ml) with strain L1–82 showing intermediate sensitivity. This is consistent with the lack of *erm*(B) in these five isolates. MIC tests confirmed the sensitivity of the four isolates to erythromycin, while isolate L1–82, although lacking a defined *erm*(B) gene, again showed intermediate sensitivity with some breakthrough growth occurring up to 250 μg/ml. The only other resistances observed were to the antimicrobials for which anaerobic bacteria often have intrinsic resistance, namely Streptomycin, Kanamycin and Ciprofloxacin.

**Table 3.** Antimicrobial resistance profile of *Roseburia intestinalis* strains.

Antibiotic/Strain	M50/1	L1–82	XB6B4	PC335	PC352
Amp (10 µg/ml)	S	S	S	S	S
Van (30 µg/ml)	S	S	S	S	S
Strep* (10 µg/ml)	IS*	IS*	R*	R*	R*
Kan* (30 µg/ml)	IS*	IS*	IS*	IS*	R*
CN (30 µg/ml)	S	S	S	S	S
Erm (15 µg/ml)	S	IS	S	S	S
Tet (30 µg/ml)	S	R	IS	IS	R
Cip* (10 µg/ml)	IS*	IS*	R*	R*	R*
MTZ (5 µg/ml)	S	S	S	S	S
Chl (30 µg/ml)	S	S	S	S	S
DA (10 µg/ml)	S	S	S	S	S

*Intrinsic resistance reported in anaerobic bacteria.

Phenotypes based on the diameter of zones (mm) indicating sensitive are as follows: Resistance (R, 8–10 mm), intermediate susceptibility (IS, 11–15 mm), and susceptibility (>15 mm). Amp – Ampicillin; Van – Vancomycin; Strep – Streptomycin; Kan – Kanamycin; CN – Gentamycin; Erm – Erythromycin; Tet – Tetracycline; Cip – Ciprofloxacin; MTZ – Metronidazole; Chl – Chloramphenicol; DA – Clindamycin.

### Sporulation capability in R. intestinalis

A core set of approximately 60 genes encompassing key genes involved in various stages of spore formation, maturation, and eventual germination governs the complex process of sporulation within the Bacillota phylum in the human gut.^55,58,59^ These genes include, but are not limited to, *spo0A* as a master regulator of sporulation initiation, *sigE, sigF, sigG* and *sigK* responsible for directing RNA polymerase toward sporulation-specific genes, and *cotH, cotY*, and *cotA* which contribute to spore coat assembly. Additionally, genes like *gerA, gerB*, and *gerC* associated with germination are vital for the spore’s transition back into an active vegetative state.

The sporulation signature genes had significant hits in the *R. intestinalis* pangenome. Of these, 38 sporulation related genes were identified in the core genome across all 16 genomes and another seven were present in all but one strain (Supplementary Table S6). The master regulator *spo0A* gene along with all the key sporulation specific RNA polymerase sigma factors (*sigE, sigF, sigG* and *sigK)* were within the core genome for all 16 *R. intestinalis* genomes except that strain AM37-1AC apparently lacked the *spo0A* gene. The majority of the conserved core sporulation genes described in other Bacillota were also present in the *R. intestinalis* genomes.^55,58,59^ A list of sporulation genes identified for the representative *R. intestinalis* strain PC352 is given in Table 4.

**Table 4.** Sporulation gene signatures in *R. intestinalis* and other spore forming bacteria.

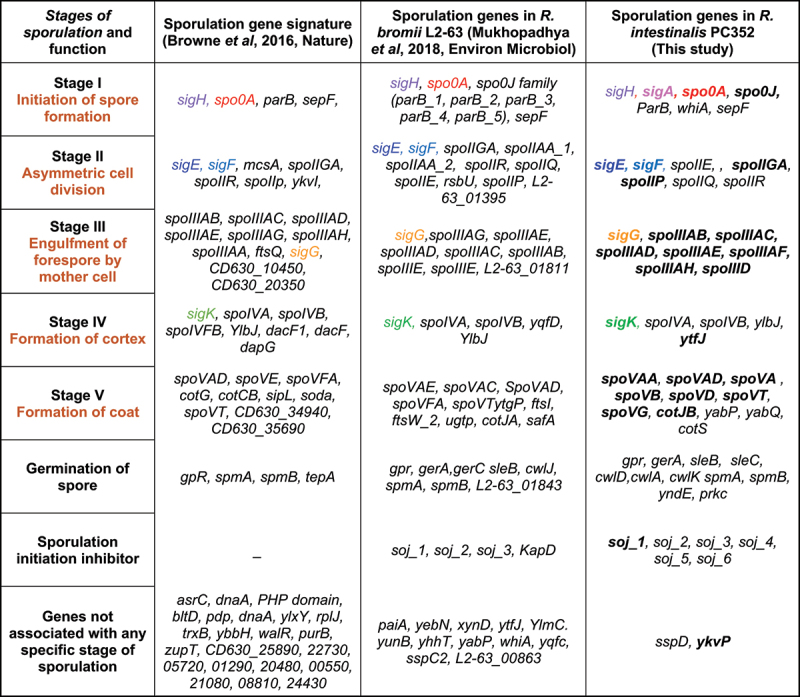

**Figure 7.**
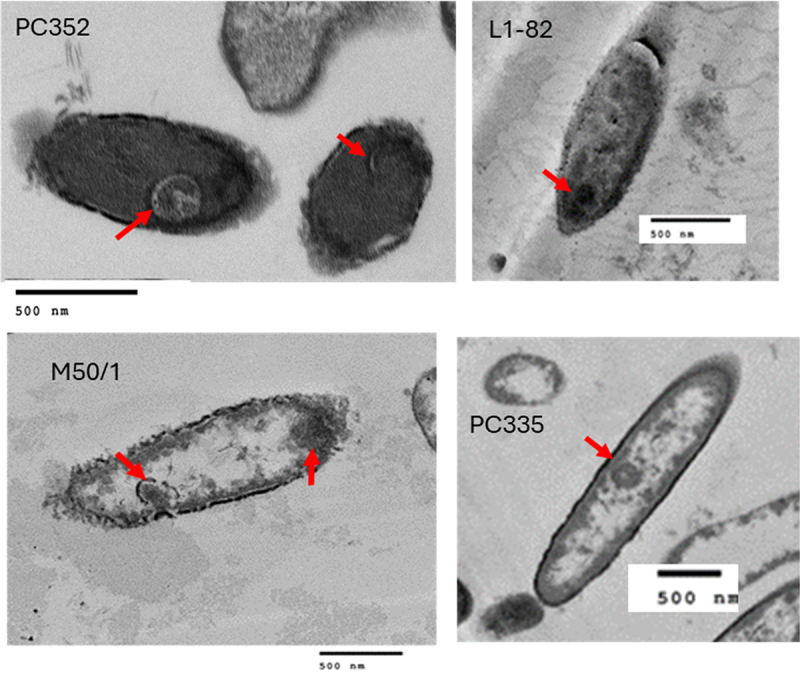
Transmission electron microscopy images showing spores present (red arrows) in individual cells of four *R. intestinalis* strains.

We tested the actual sporulation capability of *R. intestinalis* strains (L1–82, M50/1, PC335 and PC352) in laboratory tests. *R. intestinalis* cultures grown for 72 h were subjected to heat-shock and oxygen exposure to induce stress and attempt to trigger sporulation. Subsequently, cells were fixed and examined by TEM. Spores are clearly visible at the magnifications shown (Figure 7). Further work would be required to ascertain whether strains AM36-10AT and BIOML-A1, which contain the same 59 sporulation genes, also sporulate, and also the minimum number of genes required to enable sporulation.

### Flagella and motility

Flagellum synthesis also requires multiple genes, which have been shown to be present in three motility gene loci in *R. intestinalis* L1–82.^60^ All of the required genes were part of the core genome in all 16 *R. intestinalis* strains demonstrating their ability to construct complete flagella (Supplementary Table S7). The motility of strains PC335 and PC352 was confirmed using hanging drop method and light microscopy of live cells. A complete set of chemotaxis genes was identified in the core genome (Supplementary Table S8), adjacent to one of the motility loci, indicating the co-dependence of these two bacterial traits.

## Discussion

*Roseburia intestinalis* is an important butyrate producing bacterial species, abundant in the human large intestine, with potential beneficial effects in inflammatory bowel disease, colorectal cancer, atherosclerosis and metabolic syndrome, and also a possible role as a next generation probiotic.^29,61^ This study is the first specific pangenome analysis of *R. intestinalis* using 16 known human isolates of *R. intestinalis* to provide a comprehensive assessment of the metabolic function of this species. The genome analysis showed clustering of strains in two clades from Asia and Europe with a possible further American clade, illustrating potential geographical stratification. This type of stratification has been demonstrated and studied extensively in bacteria such as *Helicobacter pylori* where phenotypic and genotypic differences have been noted among bacterial strains from large datasets from different continents, reflecting evolutionary dynamics, human diet, and human migration.^62^

The genomes of the 16 *R. intestinalis* strains encoded between 97 and 123 glycoside hydrolases (GHs), more than the 70 previously reported.^28^ Five of these GHs were conserved across all strains in the core genome and were involved in starch, xylan, and mannose utilization. Growth assays confirmed the functionality of these genes in the five strains tested, and the presence of these GHs is consistent with earlier reports on substrate utilization of these bacteria.^21,63^ The combination of substrate binding and GH domains in multi-domain representatives of these genes in all isolates further indicates the importance of starch, xylan and mannose as key substrates for *R. intestinalis* growth and survival in the large intestine. There was no obvious reason from the genome information for the poorer ability of strains PC335 and PC352 to utilize the potato and corn starch for growth compared to other isolates.

The large number of CAZymes in the genomes, frequently present in multifunctional gpPULs, also highlights the dependence of these bacteria on variable substrate degradation, facilitating cross-feeding of other bacterial strains in the gut lumen and maintaining a healthy gut ecology.^12,57^ Studies like this, identifying and confirming key growth substrates are also crucial in determining potential prebiotic and dietary approaches to enhance populations of these important resident bacteria *in vivo*.

Strain-specific growth on rhamnose was unique to the most recently isolated PC335 strain. This was also the only cultured strain containing the unique rhamnose gpPUL 2. The other cultured strains (and indeed all the other strains) all contained rhamnose gpPUL 1, which was also identified as mannose gpPUL 1 and may thus be involved in mannose utilization. It is possible that rhamnose gpPUL 2 is present in strain PC335 (and strains AM37-1AC and SNUG30017) that contains two GH78 enzymes, three regulatory proteins and a chemotaxis protein is more specific for rhamnose than the other GH78 enzymes detected during the genome analysis. Growth on rhamnose only occurred after the strain was repeatedly subcultured on this substrate, implying that this is an inducible trait. The related bacterium, *Roseburia inulinivorans*, was previously shown to differentially express genes for fucose utilization, accompanied by a shift to propionate production,^64^ although propionate was not detected following growth of strain PC335 on rhamnose. The presence of these specific CAZy domains highlights the adaptive growth capabilities of these bacteria to target rhamnose, a less common but nutritionally relevant carbohydrate, as part of their metabolic strategies. This novel finding may reflect the current use of rhamnose as a sweetener and anti-obesity agent and indicate bacterial evolution following shifts in human dietary predilections in recent years.^65^

In the set of *R. intestinalis* strains analyzed in this study, tetracycline resistance genes were widespread, identified in 10 out of 16 strains. All these genes were present in the shell of the pangenome, and some were in close proximity to transposon coding gene *tnpV*, and other mobility genes suggesting that they were acquired horizontally from other bacterial species. Tetracycline resistance *tet*(40) genes were located in tandem with *tet*(O) in four strains, including three of the six Asian strains. *Erm*(B) was only identified in three strains, all Asian, two of which also included *tet*(O) and *tet*(40). This higher presence of resistance genes could reflect a more widespread use of antibiotics in these populations, although this, in addition to determining whether geographical clustering is linked to dietary differences, could only be confirmed if associated metadata was available. A comparative analysis of genes flanking the *tet*(W) resistance genes in various anaerobic gut bacteria including *Roseburia hominis* A2–183 also suggested the presence of conserved sequences potentially comprising a mobile mini-element, indicating the role of horizontal gene transfer in the acquisition of tetracycline resistance genes.^66^

Antibiotic sensitivity testing confirmed that the four strains available were all able to grow in the presence of tetracycline while strain M50/1 (lacking the resistance genes) was sensitive to tetracycline. The observed MIC ranged from 16 to 128 ug/ml with co-carriage of *tet*(40) not appearing to confer additional resistance. A separate study on *R. intestinalis* L1–82 strain also identified *tet*(O) and *tet*(40) resistance genes but, in contrast to our data, these authors deemed the strain sensitive to tetracycline and erythromycin using the disk diffusion method.^29^ This highlights the challenges in assessing antibiotic resistance in bacterial strains and is especially pertinent as an expert panel have listed *Roseburia* as one of several probiotic candidate species.^67^ The identification of functional tetracycline resistance genes, potentially encoded on mobile genetic elements, in multiple but not all strains indicate the importance of careful evaluation of individual strains for their suitability as probiotic and live-biotherapeutic candidates to minimize the risk of transferring resistance to other resident gut bacterial populations. Understanding the role of commensal bacteria as reservoirs of (potentially transferable) antimicrobial resistance genes provides critical information for global antimicrobial stewardship.

This study confirmed the presence of flagellin proteins and mobility phenotype in these strains. Flagella are also immunomodulatory molecules, and purified flagellin proteins from a different *Roseburia* species (*R. inulinivorans* A2–194) and the related bacterium *Eubacterium rectale* A1–86 stimulated secretion of the pro-inflammatory cytokine IL-8.^68^ However, motility is also an important feature for successful colonization and substrate utilization within the gut ecosystem, further emphasized by the co-location of chemotaxis genes adjacent to the flagellin synthesis genes. Flagellin proteins in *R. inulinivorans* A2–194 were upregulated during growth on starch compared to inulin.^69^

Another important finding of this study was the detection of a sporulation signature in the genomes of all 16 *R. intestinalis* strains with the majority of the genes present in the core genome. This suggests that spore formation is an intrinsic trait of this species and we confirmed the presence of spores in strain four strains by transmission electron microscopy. The *spoOA* gene was not detected in strain AM37-1AC, but this may be due to the relative incompleteness of this genome sequence. Spore formation in this anaerobic species can explain their environmental survival, host-to-host transmission and inheritability within families.^70^ The ability to form spores has significant implications in potential marketing of *R. intestinalis* strains as probiotics, providing alternative delivery options than utilization of viable anaerobic bacteria.

This comprehensive assessment of *R. intestinalis* strains through both genotypic and phenotypic characterization with specific focus on antimicrobial resistance, carbohydrate utilization and sporulation has shown distinct geographical evolution and gene acquisition profiles. Our results demonstrate that together, sporulation and specific dietary components (namely xylan, starch and mannose) are key factors in maintaining populations of these important butyrate producers in the human gut microbiota. This study also underscores the critical importance of validating phenotypic characteristics inferred from genomic information and emphasizes that the mere presence of genes does not guarantee their functionality. Ultimately, this improved understanding of carbohydrate utilization and sporulation in this important group of butyrate producing bacteria has significant implications for both dietary and probiotic interventions aimed at enhancing gut health.

## Supplementary Material

Supplemental Material

## Data Availability

This Whole Genome Shotgun project has been deposited at DDBJ/ENA/GenBank under the accession JAFEVC000000000 and JAFEVD000000000. The version described in this paper is version XXXXXX010000000.
