# Supplementary material for: Novel insights into carbohydrate utilisation, antimicrobial resistance, and sporulation potential in Roseburia intestinalis isolates across diverse geographical locations
Source: Gut Microbes. 2025 Mar 16;17(1):2473516. doi: 10.1080/19490976.2025.2473516 (PMC11913394; doi:10.1080/19490976.2025.2473516)
Supplement: Supplemental Material [file KGMI_A_2473516_SM2914.zip › Supplementary_material_revision_clean.docx]

**Supplementary Table S1.** Proportion of *Roseburia intestinalis* genes assigned to each COG functional category within the core, shell and cloud genomes.

| **Category** | **Description** | **Core %** | **Shell %** | **Cloud %** |
| --- | --- | --- | --- | --- |
| C | Energy production and conversion | 6.03% | 2.11% | 1.57% |
| D | Cell cycle control, cell division, chromosome partitioning | 3.38% | 3.37% | 4.00% |
| E | Amino acid transport and metabolism | 11.03% | 5.20% | 3.57% |
| F | Nucleotide transport and metabolism | 4.93% | 2.25% | 1.13% |
| G | Carbohydrate transport and metabolism | 9.93% | 9.55% | 3.74% |
| H | Coenzyme transport and metabolism | 4.49% | 1.12% | 1.22% |
| I | Lipid transport and metabolism | 3.38% | 1.40% | 1.31% |
| J | Translation, ribosomal structure and biogenesis | 13.38% | 4.63% | 3.13% |
| K | Transcription | 7.65% | 12.92% | 16.10% |
| L | Replication, recombination and repair | 3.68% | 13.62% | 14.27% |
| M | Cell wall/membrane/envelope biogenesis | 4.85% | 6.74% | 7.57% |
| N | Cell motility | 1.47% | 2.81% | 2.09% |
| O | Posttranslational modification, protein turnover, chaperones | 3.38% | 1.69% | 1.74% |
| P | Inorganic ion transport and metabolism | 3.31% | 1.97% | 1.65% |
| Q | Secondary metabolites biosynthesis, transport and catabolism | 0.15% | 0.98% | 0.87% |
| R | General function prediction only | 6.03% | 5.48% | 4.18% |
| S | Function unknown | 2.65% | 3.23% | 1.57% |
| T | Signal transduction mechanisms | 5.88% | 8.43% | 7.31% |
| U | Intracellular trafficking, secretion, and vesicular transport | 1.25% | 2.67% | 3.74% |
| V | Defense mechanisms | 2.87% | 4.63% | 6.44% |
| W | Extracellular structures | 0.07% | 0.14% | 0.26% |
| X | Mobilome: prophages, transposons | 0.00% | 4.92% | 12.53% |
| Z | Cytoskeleton | 0.22% | 0.14% | 0.00% |
| **TOTAL NUMBER OF GENES WITH HITS > 70% IDENTITY** | | 1173 | 607 | 974 |

**Supplementary Table S2.** Proportion of genes assigned to each COG functional category within the unique genes of each genome. (*Number of genes with blast hits with >70% percentage identity highlighted). Category description as in Supplementary Table 1.

| **Category** | **2789STDY5834960** | **AF31-21AC** | **AF36-10AT** | **AM22-21LB** | **AM37-1AC** | **AM43-11** | **BIOML-A1** | **BSD2780061689_150309_G12** | **J1101437_171009_C5** | **L1-82** | **M50_1** | **MSK_17_84** | **PC335** | **PC352** | **SNUG30017** | **XB6B4** |
| --- | --- | --- | --- | --- | --- | --- | --- | --- | --- | --- | --- | --- | --- | --- | --- | --- |
| C | 3.6% | 0.0% | 0.0% | 1.8% | 0.0% | 0.0% | 9.1% | 0.0% | 4.8% | 2.3% | 7.1% | 0.0% | 0.0% | 1.7% | 0.0% | 0.0% |
| D | 3.6% | 7.4% | 0.0% | 3.6% | 13.0% | 0.0% | 9.1% | 3.9% | 4.8% | 2.3% | 4.3% | 5.5% | 4.2% | 1.7% | 0.0% | 3.6% |
| E | 0.0% | 0.0% | 0.0% | 3.6% | 8.7% | 0.0% | 0.0% | 0.0% | 3.2% | 4.7% | 8.6% | 0.0% | 1.4% | 2.5% | 5.0% | 7.3% |
| F | 0.0% | 1.9% | 33.3% | 0.0% | 0.0% | 0.0% | 9.1% | 0.0% | 1.6% | 0.0% | 0.0% | 0.0% | 0.0% | 1.7% | 0.0% | 0.0% |
| G | 3.6% | 3.7% | 0.0% | 3.6% | 0.0% | 2.6% | 0.0% | 3.9% | 3.2% | 2.3% | 2.9% | 1.8% | 1.4% | 4.2% | 0.0% | 3.6% |
| H | 0.0% | 1.9% | 0.0% | 3.6% | 0.0% | 0.0% | 0.0% | 0.0% | 0.0% | 0.0% | 0.0% | 1.8% | 0.0% | 2.5% | 5.0% | 0.0% |
| I | 1.8% | 0.0% | 0.0% | 0.0% | 4.3% | 0.0% | 0.0% | 3.9% | 1.6% | 1.2% | 5.7% | 0.0% | 0.0% | 0.0% | 0.0% | 0.0% |
| J | 3.6% | 1.9% | 0.0% | 1.8% | 4.3% | 5.1% | 9.1% | 2.0% | 3.2% | 1.2% | 8.6% | 1.8% | 1.4% | 2.5% | 5.0% | 3.6% |
| K | 28.6% | 11.1% | 0.0% | 10.7% | 13.0% | 38.5% | 9.1% | 19.6% | 7.9% | 17.4% | 12.9% | 12.7% | 18.1% | 14.2% | 10.0% | 21.8% |
| L | 17.9% | 24.1% | 0.0% | 23.2% | 13.0% | 25.6% | 9.1% | 17.6% | 11.1% | 15.1% | 7.1% | 7.3% | 15.3% | 16.7% | 5.0% | 14.5% |
| M | 8.9% | 5.6% | 33.3% | 1.8% | 13.0% | 2.6% | 0.0% | 9.8% | 6.3% | 3.5% | 11.4% | 16.4% | 11.1% | 3.3% | 5.0% | 5.5% |
| N | 0.0% | 0.0% | 0.0% | 3.6% | 4.3% | 0.0% | 0.0% | 3.9% | 3.2% | 0.0% | 2.9% | 1.8% | 2.8% | 0.0% | 0.0% | 1.8% |
| O | 0.0% | 0.0% | 0.0% | 1.8% | 0.0% | 2.6% | 0.0% | 2.0% | 3.2% | 0.0% | 0.0% | 1.8% | 1.4% | 3.3% | 0.0% | 0.0% |
| P | 0.0% | 3.7% | 0.0% | 0.0% | 0.0% | 2.6% | 0.0% | 2.0% | 4.8% | 0.0% | 1.4% | 0.0% | 1.4% | 2.5% | 0.0% | 1.8% |
| Q | 3.6% | 1.9% | 0.0% | 0.0% | 0.0% | 0.0% | 18.2% | 0.0% | 0.0% | 1.2% | 1.4% | 0.0% | 0.0% | 0.0% | 5.0% | 1.8% |
| R | 5.4% | 0.0% | 0.0% | 3.6% | 0.0% | 2.6% | 0.0% | 2.0% | 0.0% | 5.8% | 5.7% | 7.3% | 5.6% | 3.3% | 15.0% | 3.6% |
| S | 0.0% | 3.7% | 0.0% | 3.6% | 0.0% | 0.0% | 0.0% | 3.9% | 3.2% | 2.3% | 0.0% | 1.8% | 1.4% | 0.8% | 5.0% | 0.0% |
| T | 3.6% | 9.3% | 0.0% | 3.6% | 17.4% | 2.6% | 9.1% | 5.9% | 15.9% | 3.5% | 14.3% | 14.5% | 4.2% | 2.5% | 0.0% | 10.9% |
| U | 1.8% | 0.0% | 0.0% | 3.6% | 0.0% | 0.0% | 0.0% | 3.9% | 7.9% | 1.2% | 0.0% | 7.3% | 5.6% | 3.3% | 5.0% | 0.0% |
| V | 7.1% | 13.0% | 0.0% | 5.4% | 4.3% | 2.6% | 9.1% | 11.8% | 4.8% | 3.5% | 1.4% | 7.3% | 8.3% | 5.8% | 10.0% | 18.2% |
| W | 0.0% | 0.0% | 0.0% | 0.0% | 0.0% | 0.0% | 0.0% | 0.0% | 1.6% | 0.0% | 0.0% | 3.6% | 0.0% | 0.0% | 0.0% | 0.0% |
| X | 7.1% | 11.1% | 33.3% | 21.4% | 4.3% | 12.8% | 9.1% | 3.9% | 7.9% | 32.6% | 4.3% | 7.3% | 16.7% | 27.5% | 25.0% | 1.8% |
| Z | 0.0% | 0.0% | 0.0% | 0.0% | 0.0% | 0.0% | 0.0% | 0.0% | 0.0% | 0.0% | 0.0% | 0.0% | 0.0% | 0.0% | 0.0% | 0.0% |
| **Genes*** | 51 | 48 | 3 | 49 | 20 | 37 | 11 | 44 | 53 | 68 | 59 | 44 | 67 | 95 | 18 | 47 |

**Supplementary Table S3.** Proportion of genes assigned to each COG functional category in the exclusively absent genes of each genome**.** (*Number of genes with blast hits with >70% percentage identity highlighted)

| **Category** | **2789STDY5834960** | **AF31-21AC** | **AF36-10AT** | **AM22-21LB** | **AM37-1AC** | **AM43-11** | **BIOML-A1** | **BSD2780061689_150309_G12** | **J1101437_171009_C5** | **L1-82** | **M50_1** | **MSK_17_84** | **PC335** | **PC352** | **SNUG30017** | **XB6B4** |
| --- | --- | --- | --- | --- | --- | --- | --- | --- | --- | --- | --- | --- | --- | --- | --- | --- |
| C | 0.0% | 0.0% | 0.0% | 7.1% | 0.0% | 0.0% | 0.0% | 0.0% | 0.0% | 0.0% | 0.0% | 0.0% | 0.0% | 0.0% | 0.0% | 0.0% |
| D | 0.0% | 0.0% | 0.0% | 0.0% | 0.0% | 0.0% | 0.0% | 0.0% | 0.0% | 0.0% | 0.0% | 0.0% | 0.0% | 10.0% | 0.0% | 13.3% |
| E | 0.0% | 20.0% | 0.0% | 28.6% | 10.0% | 0.0% | 0.0% | 0.0% | 0.0% | 0.0% | 7.7% | 0.0% | 0.0% | 20.0% | 0.0% | 13.3% |
| F | 0.0% | 0.0% | 0.0% | 0.0% | 0.0% | 0.0% | 100.0% | 0.0% | 0.0% | 0.0% | 0.0% | 20.0% | 0.0% | 0.0% | 0.0% | 0.0% |
| G | 0.0% | 0.0% | 0.0% | 0.0% | 10.0% | 0.0% | 0.0% | 0.0% | 0.0% | 0.0% | 7.7% | 0.0% | 66.7% | 0.0% | 100.0% | 6.7% |
| H | 0.0% | 20.0% | 0.0% | 7.1% | 0.0% | 0.0% | 0.0% | 0.0% | 0.0% | 0.0% | 7.7% | 0.0% | 0.0% | 0.0% | 0.0% | 0.0% |
| I | 0.0% | 0.0% | 0.0% | 7.1% | 0.0% | 0.0% | 0.0% | 0.0% | 0.0% | 0.0% | 15.4% | 0.0% | 0.0% | 0.0% | 0.0% | 0.0% |
| J | 0.0% | 0.0% | 0.0% | 0.0% | 10.0% | 0.0% | 0.0% | 20.0% | 0.0% | 0.0% | 15.4% | 0.0% | 0.0% | 30.0% | 0.0% | 13.3% |
| K | 0.0% | 0.0% | 0.0% | 0.0% | 20.0% | 66.7% | 0.0% | 20.0% | 0.0% | 0.0% | 15.4% | 20.0% | 33.3% | 0.0% | 0.0% | 20.0% |
| L | 100.0% | 0.0% | 0.0% | 0.0% | 10.0% | 33.3% | 0.0% | 20.0% | 0.0% | 0.0% | 7.7% | 0.0% | 0.0% | 20.0% | 0.0% | 6.7% |
| M | 0.0% | 0.0% | 0.0% | 0.0% | 0.0% | 0.0% | 0.0% | 0.0% | 100.0% | 0.0% | 0.0% | 0.0% | 0.0% | 0.0% | 0.0% | 0.0% |
| N | 0.0% | 20.0% | 0.0% | 0.0% | 0.0% | 0.0% | 0.0% | 0.0% | 0.0% | 0.0% | 0.0% | 0.0% | 0.0% | 0.0% | 0.0% | 6.7% |
| O | 0.0% | 20.0% | 0.0% | 0.0% | 0.0% | 0.0% | 0.0% | 0.0% | 0.0% | 0.0% | 0.0% | 0.0% | 0.0% | 10.0% | 0.0% | 0.0% |
| P | 0.0% | 20.0% | 0.0% | 14.3% | 0.0% | 0.0% | 0.0% | 20.0% | 0.0% | 0.0% | 0.0% | 20.0% | 0.0% | 0.0% | 0.0% | 0.0% |
| Q | 0.0% | 0.0% | 0.0% | 0.0% | 0.0% | 0.0% | 0.0% | 0.0% | 0.0% | 0.0% | 7.7% | 0.0% | 0.0% | 0.0% | 0.0% | 0.0% |
| R | 0.0% | 0.0% | 0.0% | 7.1% | 0.0% | 0.0% | 0.0% | 20.0% | 0.0% | 0.0% | 0.0% | 0.0% | 0.0% | 0.0% | 0.0% | 6.7% |
| S | 0.0% | 0.0% | 0.0% | 14.3% | 10.0% | 0.0% | 0.0% | 0.0% | 0.0% | 0.0% | 0.0% | 0.0% | 0.0% | 0.0% | 0.0% | 0.0% |
| T | 0.0% | 0.0% | 0.0% | 14.3% | 30.0% | 0.0% | 0.0% | 0.0% | 0.0% | 0.0% | 0.0% | 40.0% | 0.0% | 10.0% | 0.0% | 13.3% |
| U | 0.0% | 0.0% | 0.0% | 0.0% | 0.0% | 0.0% | 0.0% | 0.0% | 0.0% | 0.0% | 0.0% | 0.0% | 0.0% | 0.0% | 0.0% | 0.0% |
| V | 0.0% | 0.0% | 0.0% | 0.0% | 0.0% | 0.0% | 0.0% | 0.0% | 0.0% | 0.0% | 15.4% | 0.0% | 0.0% | 0.0% | 0.0% | 0.0% |
| W | 0.0% | 0.0% | 0.0% | 0.0% | 0.0% | 0.0% | 0.0% | 0.0% | 0.0% | 0.0% | 0.0% | 0.0% | 0.0% | 0.0% | 0.0% | 0.0% |
| X | 0.0% | 0.0% | 0.0% | 0.0% | 0.0% | 0.0% | 0.0% | 0.0% | 0.0% | 0.0% | 0.0% | 0.0% | 0.0% | 0.0% | 0.0% | 0.0% |
| Z | 0.0% | 0.0% | 0.0% | 0.0% | 0.0% | 0.0% | 0.0% | 0.0% | 0.0% | 0.0% | 0.0% | 0.0% | 0.0% | 0.0% | 0.0% | 0.0% |
| **Genes*** | 1 | 4 | 0 | 10 | 9 | 3 | 1 | 3 | 1 | 0 | 11 | 4 | 3 | 10 | 3 | 12 |

**Supplementary Figure S1.** Proportion of *Roseburia intestinalis* genes assigned to each COG functional category in the core, shell and cloud genomes.

**
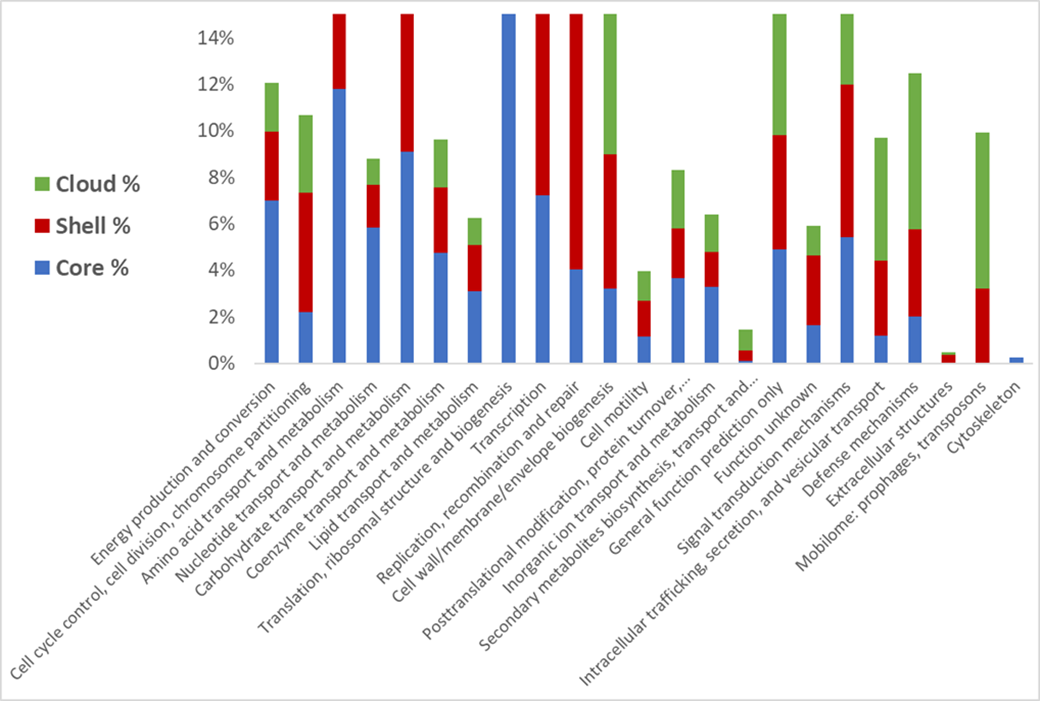
**

**Supplementary Figure S2.** Proportion of genes assigned to each COG functional category in the unique genes of each genomes.

**Supplementary Figure S3.** Proportion of genes assigned to each COG functional category in the exclusively absent genes of each genome.


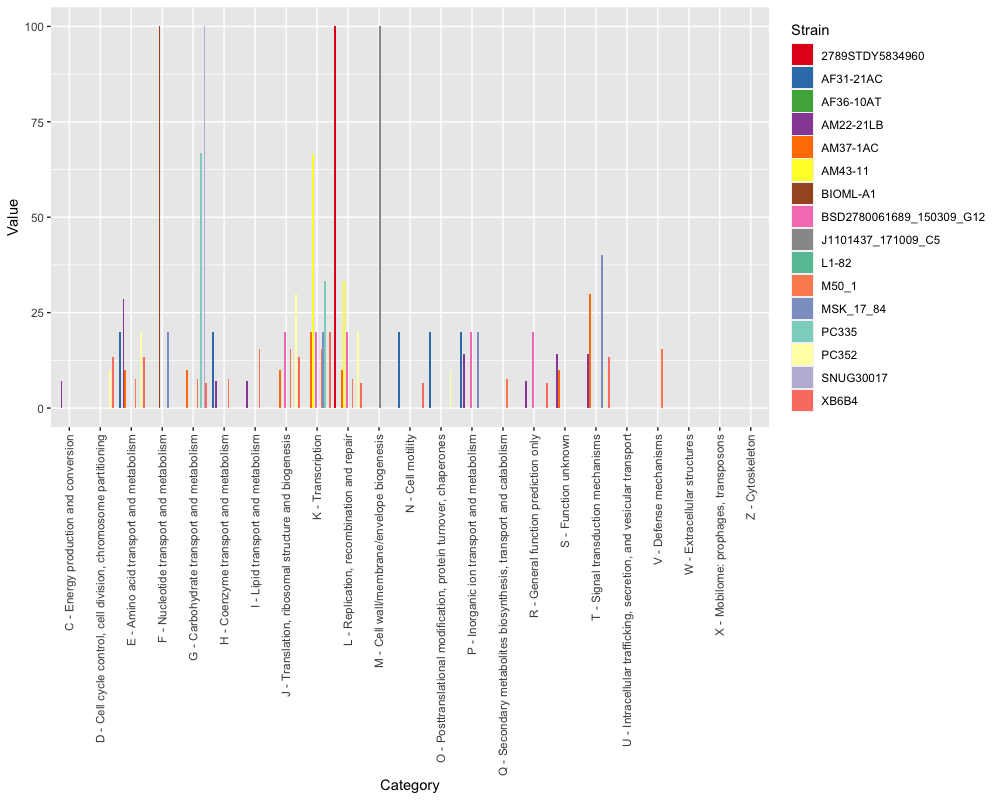


**Supplementary Figure S4.** Percentage of each glycoside hydrolase domain.


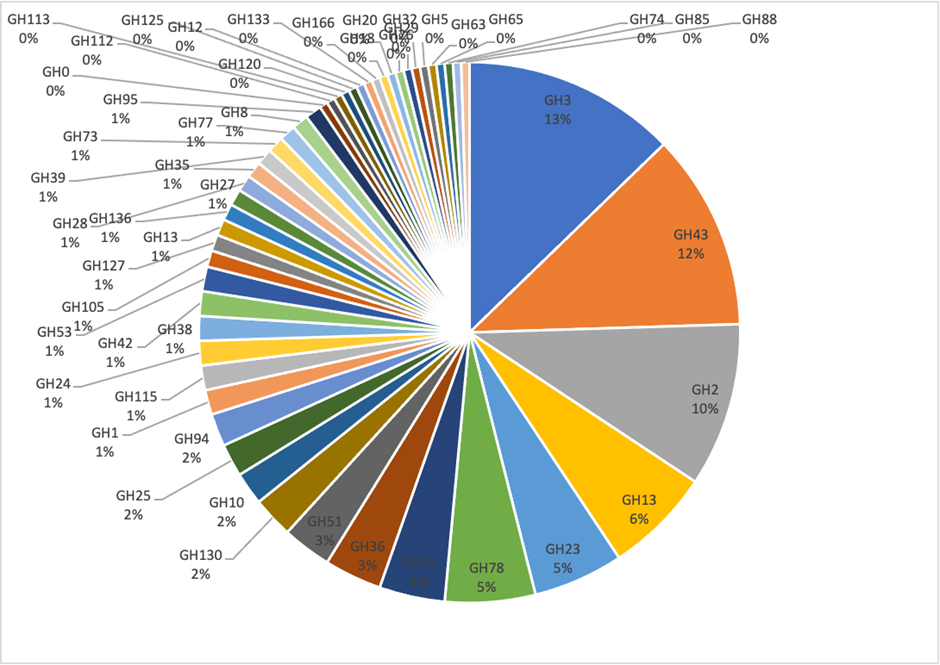


**Supplementary Figure S5.** *Roseburia intestinalis* predicted starch utilisation gpPULs schematic representation.

**
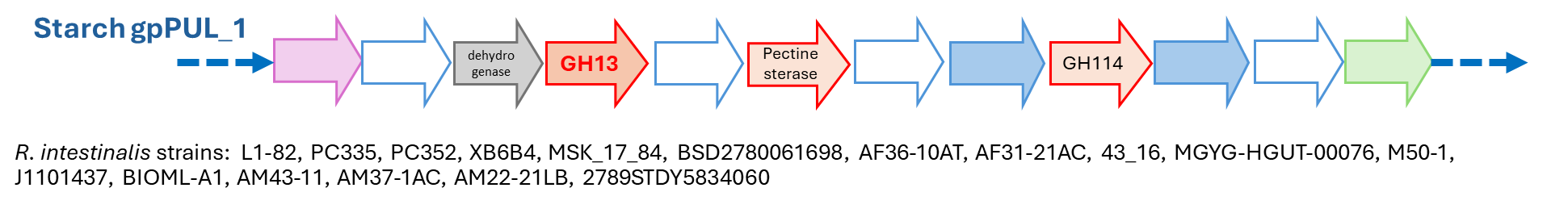
**

**
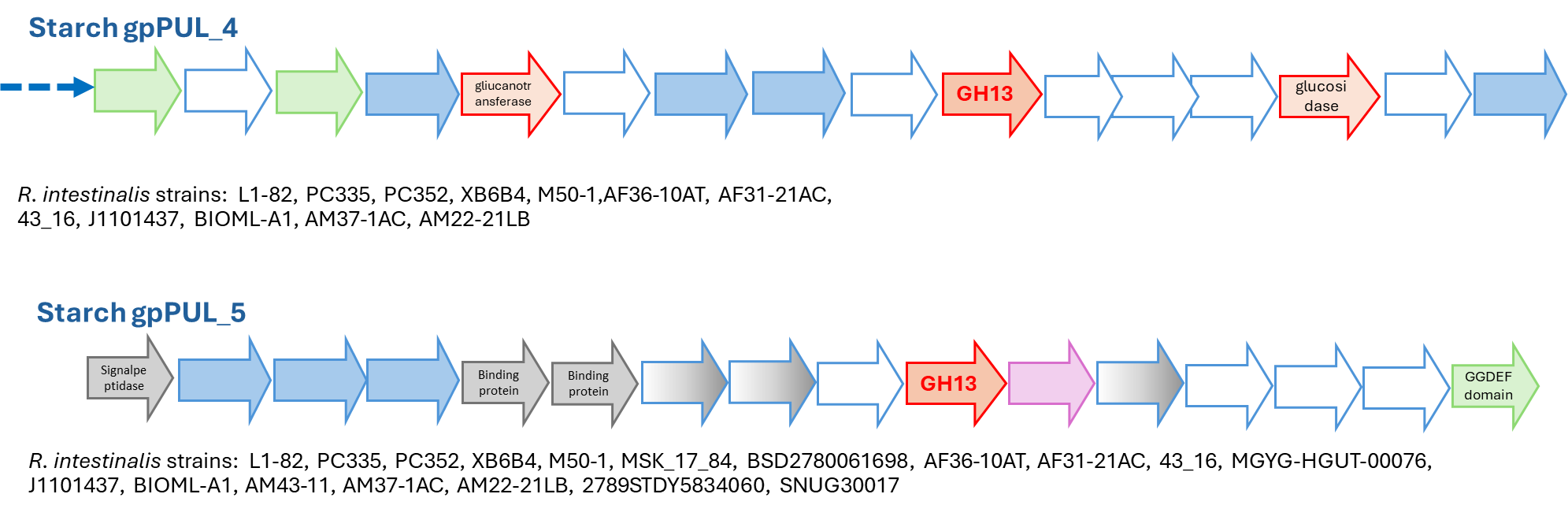

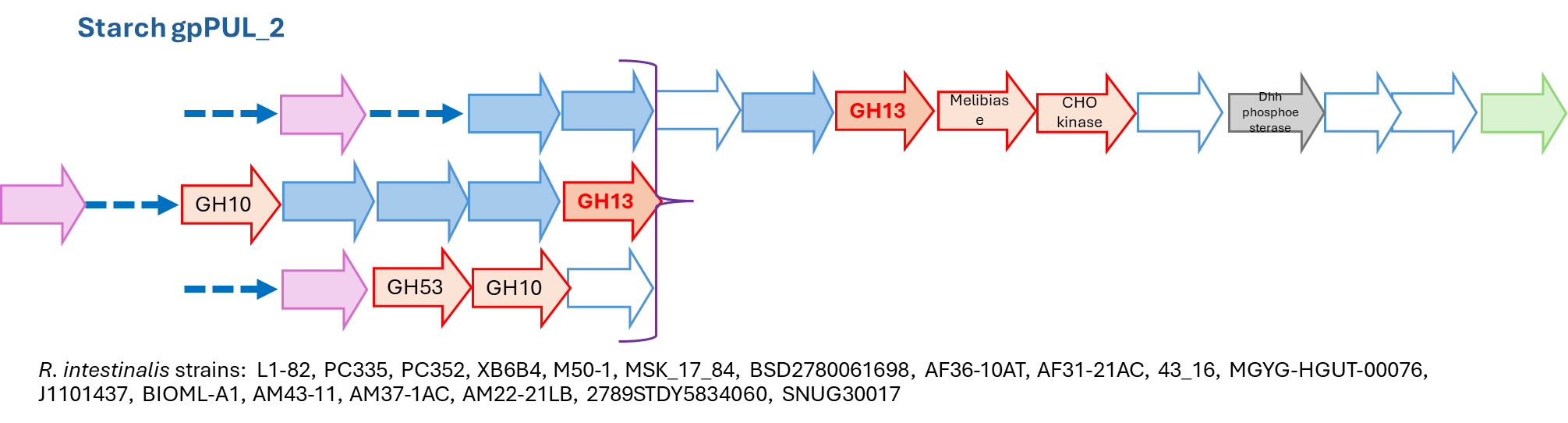

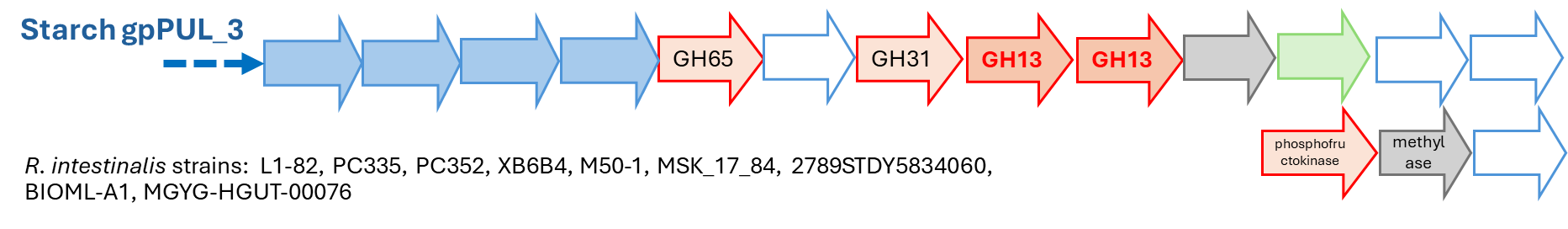

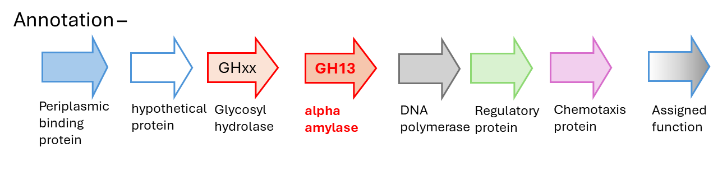
**

**Supplementary Table S4.** AMR genes identified by RGI comparison to CARD.

| **Consensus Gene Identifier** | **AMR Gene Hit** | **AMR Gene Description** | **Pangenome Set (number of genomes)** | **Genomes Present In** |
| --- | --- | --- | --- | --- |
| 2789STDY5834960_00368 | MCR-8.1 | MCR phosphoethanolamine transferase | Shell (11) | 2789STDY5834960  AF31-21AC  AF36-10AT  AM22-21LB  AM37-1AC  BIOML-A1  BSD2780061689_150309_G12  L1-82  M50_1  PC352  SNUG30017 |
| 2789STDY5834960_03848 | mupB | Antibiotic resistant isoleucyl-tRNA synthetase (ileS) | Cloud (1) | 2789STDY5834960 |
| AF31-21AC_02025 | ErmB | Erm 23S ribosomal RNA methyltransferase | Shell (3) | AF31-21AC  AM37-1AC  AM43-11 |
| AF31-21AC_02216 | tetO | Tetracycline-resistant ribosomal protection protein | Shell (3) | AF31-21AC  BSD2780061689_150309_G12  XB6B4 |
| AF31-21AC_02217 | tet(40) | Major facilitator superfamily (MFS) antibiotic efflux pump | Shell (4) | AF31-21AC  AM22-21LB  AM43-11  L1-82 |
| AF31-21AC_03212 | mdtG | Major facilitator superfamily (MFS) antibiotic efflux pump | Shell (9) | AF31-21AC  AF36-10AT  AM22-21LB  AM37-1AC  BIOML-A1  BSD2780061689_150309_G12  L1-82  PC352  XB6B4 |
| AF36-10AT_01143 | tet(O) | Tetracycline-resistant ribosomal protection protein | Shell (7) | AF36-10AT  AM22-21LB AM43-11  BIOML-A1  L1-82  PC335  PC352 |
| AM22-21LB_01735 | AAC(6')-Ie-APH(2'')-Ia | APH(2''); AAC(6') | Cloud (2) | AM22-21LB  AM43-11 |
| AM43-11_03394 | MCR-8.1 | MCR phosphoethanolamine transferase | Shell (3) | AM43-11  J1101437_171009_C5  MSK_17_84 |

**Supplementary Figure S6.** *R. intestinalis* pangenome Tet() and associated mobile genetic elements.

**Supplementary Table S5.** Significant hits against the ISFinder database identified with blastp

| **Consensus Gene Identifier** | **ISFinder Hit** | **ISFinder Description** | **Pangenome Set (number of genomes)** | **Genomes Present In** |
| --- | --- | --- | --- | --- |
| 2789STDY5834960_00001 | ISDha13 | IS605 ORF Transposase | Shell (6) | 2789STDY5834960  AF36-10AT  BSD2780061689_150309_G12  L1-82  PC335  SNUG30017 |
| 2789STDY5834960_00195 | ISCb1 | ISCb1 unknown ISNCY ORF Transposase | Shell (6) | 2789STDY5834960  AF36-10AT  BIOML-A1  J1101437_171009_C5  L1-82  M50_1 |
| 2789STDY5834960_00831 | ISRin1 | ISRin1 IS5 IS5 ORF Transposase | Cloud (2) | 2789STDY5834960  L1-82 |
| 2789STDY5834960_01463 | ISDha13 | IS605 ORF Transposase | Shell (10) | 2789STDY5834960  AF36-10AT  AM22-21LB  AM43-11  BIOML-A1  BSD2780061689_150309_G12  J1101437_171009_C5  L1-82  PC352  SNUG30017 |
| 2789STDY5834960_03077 | ISDha13 | IS605 ORF Transposase | Shell (3) | 2789STDY5834960  PC352  SNUG30017 |
| 2789STDY5834960_03603 | ISClbo1 | ISClbo1 unknown IS1182 ORF Transposase | Shell (7) | 2789STDY5834960  AF31-21AC  AM43-11  BSD2780061689_150309_G12  L1-82  M50_1  PC352 |
| 2789STDY5834960_03604 | ISClbo1 | ISClbo1 unknown IS1182 ORF Transposase | Shell (6) | 2789STDY5834960  AF31-21AC  AM43-11  BSD2780061689_150309_G12  M50_1  PC352 |
| AF31-21AC_00489 | ISRgn2 | ISRgn2 unknown IS66 ORF 2 Accessory Gene | Cloud (1) | AF31-21AC |
| AF31-21AC_02026 | ISCco2 | ISCco2 ISPna2 IS1595 ORF 8 Passenger Gene | Cloud (1) | AF31-21AC |
| AF31-21AC_03268 | ISDha13 | IS605 ORF Transposase | Shell (6) | AF31-21AC  AM22-21LB  J1101437_171009_C5  MSK_17_84  PC352  XB6B4 |
| AF36-10AT_01516 | ISDha13 | IS605 ORF Transposase | Shell (9) | AF36-10AT  AM22-21LB  AM43-11  BIOML-A1  BSD2780061689_150309_G12  J1101437_171009_C5  L1-82  PC335  PC352 |
| AF36-10AT_01720 | ISCco2 | ISCco2 ISPna2 IS1595 ORF 8 Passenger Gene | Cloud (2) | AF36-10AT  BIOML-A1 |
| AF36-10AT_02390 | ISClsp1 | ISClsp1 IS150 IS3 ORF 1 Transposase | Shell (5) | AF36-10AT  AM43-11  PC335  PC352  SNUG30017 |
| AF36-10AT_03293 | ISCb1 | ISCb1 unknown ISNCY ORF Transposase | Cloud (1) | AF36-10AT |
| AM22-21LB_01735 | ISCco2 | ISCco2 ISPna2 IS1595 ORF 10 Passenger Gene | Cloud (2) | AM22-21LB  AM43-11 |
| AM22-21LB_03752 | ISClsp1 | ISClsp1 IS150 IS3 ORF 1 Transposase | Shell (6) | AM22-21LB  AM37-1AC  BSD2780061689_150309_G12  L1-82  MSK_17_84  PC335 |
| AM37-1AC_03159 | ISRin1 | ISRin1 IS5 IS5 ORF Transposase | Cloud (2) | AM37-1AC  AM43-11 |
| AM43-11_00730 | ISClte2 | ISClte2 IS605 IS200/IS605 ORF 1 Transposase | Cloud (2) | AM43-11  SNUG30017 |
| AM43-11_00824 | ISClte2 | ISClte2 IS605 IS200/IS605 ORF 1 Transposase | Cloud (2) | AM43-11  J1101437_171009_C5 |
| AM43-11_03060 | ISEnfa4 | ISEnfa4 unknown IS256 ORF Transposase | Cloud (1) | AM43-11 |
| BIOML-A1_01231 | ISDha13 | IS605 ORF Transposase | Cloud (2) | BIOML-A1  PC335 |
| BIOML-A1_01428 | ISCb1 | ISCb1 unknown ISNCY ORF Transposase | Cloud (1) | BIOML-A1 |
| L1-82_00220 | ISClbo1 | ISClbo1 unknown IS1182 ORF Transposase | Cloud (1) | L1-82 |
| L1-82_01217 | ISDha13 | IS605 ORF Transposase | Cloud (1) | L1-82 |
| L1-82_01218 | ISClsp1 | ISClsp1 IS150 IS3 ORF 1 Transposase | Cloud (2) | L1-82  PC352 |
| L1-82_02315 | ISClsp1 | ISClsp1 IS150 IS3 ORF 1 Transposase | Cloud (2) | L1-82  PC335 |
| L1-82_02422 | ISClsp1 | ISClsp1 IS150 IS3 ORF 1 Transposase | Cloud (1) | L1-82 |
| L1-82_02507 | ISClsp1 | ISClsp1 IS150 IS3 ORF 1 Transposase | Cloud (2) | L1-82  PC352 |
| L1-82_02529 | ISClsp1 | ISClsp1 IS150 IS3 ORF 1 Transposase | Cloud (1) | L1-82 |
| L1-82_02594 | ISClsp1 | ISClsp1 IS150 IS3 ORF 1 Transposase | Cloud (1) | L1-82 |
| L1-82_02598 | ISClsp1 | ISClsp1 IS150 IS3 ORF 1 Transposase | Cloud (1) | L1-82 |
| L1-82_02604 | ISClsp1 | ISClsp1 IS150 IS3 ORF 1 Transposase | Cloud (1) | L1-82 |
| L1-82_02608 | ISClsp1 | ISClsp1 IS150 IS3 ORF 1 Transposase | Cloud (1) | L1-82 |
| L1-82_03536 | ISClsp1 | ISClsp1 IS150 IS3 ORF 1 Transposase | Cloud (1) | L1-82 |
| L1-82_03989 | ISRin1 | ISRin1 IS5 IS5 ORF Transposase | Cloud (1) | L1-82 |
| PC335_02118 | ISClsp1 | ISClsp1 IS150 IS3 ORF 3 Transposase | Cloud (1) | PC335 |
| PC335_02316 | ISClsp1 | ISClsp1 IS150 IS3 ORF 1 Transposase | Cloud (1) | PC335 |
| PC335_03189 | IS655 | IS655 unknown IS3 ORF 3 Transposase | Cloud (1) | PC335 |
| PC352_00359 | ISClsp1 | ISClsp1 IS150 IS3 ORF 1 Transposase | Cloud (1) | PC352 |
| PC352_00406 | ISClsp1 | ISClsp1 IS150 IS3 ORF 1 Transposase | Cloud (1) | PC352 |
| PC352_00418 | ISClsp1 | ISClsp1 IS150 IS3 ORF 1 Transposase | Cloud (1) | PC352 |
| PC352_02225 | ISClsp1 | ISClsp1 IS150 IS3 ORF 1 Transposase | Cloud (1) | PC352 |
| PC352_02985 | ISClsp1 | ISClsp1 IS150 IS3 ORF 1 Transposase | Cloud (1) | PC352 |
| PC352_03201 | ISClsp1 | ISClsp1 IS150 IS3 ORF 1 Transposase | Cloud (1) | PC352 |
| PC352_03475 | ISClsp1 | ISClsp1 IS150 IS3 ORF 1 Transposase | Cloud (1) | PC352 |
| PC352_04085 | ISClsp1 | ISClsp1 IS150 IS3 ORF 1 Transposase | Cloud (1) | PC352 |
| PC352_04206 | ISClsp1 | ISClsp1 IS150 IS3 ORF 1 Transposase | Cloud (1) | PC352 |
| XB6B4_02207 | ISCb1 | ISCb1 unknown ISNCY ORF Transposase | Cloud (1) | XB6B4 |

**Supplementary Table S6.** Sporulation genes detected in the *R. intestinalis* pangenome.

| **Sporulation Gene Description** | **Pangenome Set** | **Genes Present In** |
| --- | --- | --- |
| Stage III sporulation protein D | Core (16) | All |
| Spore protein YkvP | Core (16) | All |
| Sporulation initiation inhibitor protein Soj | Core (16) | All |
| Chromosome-partitioning protein Spo0J | Core (16) | All |
| RNA polymerase sigma-28 factor precursor | Core (16) | All |
| CotJB protein | Core (16) | All |
| Spore protein YkvP | Core (16) | All |
| Stage V sporulation protein B | Core (16) | All |
| Spore cortex-lytic enzyme precursor | Core (16) | All |
| Putative septation protein SpoVG | Core (16) | All |
| SpoVA protein | Core (16) | All |
| Stage V sporulation protein AD | Core (16) | All |
| RNA polymerase sigma factor SigA | Core (16) | All |
| Chromosome-partitioning protein Spo0J | Core (16) | All |
| Sporulation and spore germination | Core (16) | All |
| Sporulation initiation inhibitor protein Soj | Core (16) | All |
| RNA polymerase sigma-F factor | Core (16) | All |
| SpoVA protein | Core (16) | All |
| Stage V sporulation protein AA | Core (16) | All |
| Sporulation and spore germination | Core (16) | All |
| Germination protease precursor | Core (16) | All |
| Stage II sporulation protein P (SpoIIP) | Core (16) | All |
| Sporulation initiation inhibitor protein Soj | Core (16) | All |
| Stage V sporulation protein D | Core (16) | All |
| Stage V sporulation protein D | Core (16) | All |
| Sporulation factor SpoIIGA | Core (16) | All |
| RNA polymerase sigma-E factor precursor | Core (16) | All |
| RNA polymerase sigma-F factor | Core (16) | All |
| Putative septation protein SpoVG | Core (16) | All |
| Sporulation protein YtfJ (Spore_YtfJ) | Core (16) | All |
| Sporulation initiation inhibitor protein Soj | Core (16) | All |
| Spore protein YkvP | Core (16) | All |
| stage III sporulation protein SpoAB | Core (16) | All |
| Stage III sporulation protein AC/AD protein | Core (16) | All |
| Stage III sporulation protein AC/AD protein | Core (16) | All |
| Stage III sporulation protein AE precursor | Core (16) | All |
| Stage III sporulation protein AF (Spore_III_AF) | Core (16) | All |
| SpoIIIAH-like protein | Core (16) | All |
| Putative septation protein SpoVG | Shell (15) | 2789STDY5834960, AF31-21AC, AF36-10AT, AM22-21LB, AM37-1AC, AM43-11, BIOML-A1, BSD2780061698, J1101437, L1-82, MSK_17_84, PC335, PC352, SNUG30017, XB6B4 |
| Spore protein YkvP | Shell (15) | 2789STDY5834960, AF31-21AC, AF36-10AT, AM22-21LB, AM37-1AC, AM43-11, BSD2780061698, J1101437, L1-82, M50_1, MSK_17_84, PC335, PC352, SNUG30017, XB6B4 |
| Spore cortex-lytic enzyme precursor | Shell (15) | 2789STDY5834960, AF31-21AC, AF36-10AT, AM22-21LB, AM37-1AC, AM43-11, BIOML-A1, BSD2780061698, J1101437, L1-82, M50_1, MSK_17_84, PC335, SNUG30017, XB6B4 |
| SpoVT / AbrB like domain protein | Shell (15) | 2789STDY5834960, AF31-21AC, AF36-10AT, AM22-21LB, AM37-1AC, AM43-11, BIOML-A1, BSD2780061698, L1-82, M50_1, MSK_17_84, PC335, PC352, SNUG30017, XB6B4 |
| Stage II sporulation protein E | Shell (15) | 2789STDY5834960, AF31-21AC, AF36-10AT, AM22-21LB, AM37-1AC, AM43-11, BIOML-A1, BSD2780061698, J1101437, L1-82, M50_1, MSK_17_84, PC335, PC352, XB6B4 |
| SpoIVB peptidase precursor | Shell (15) | 2789STDY5834960, AF31-21AC, AF36-10AT, AM22-21LB, AM43-11, BIOML-A1, BSD2780061698, J1101437, L1-82, M50_1, MSK_17_84, PC335, PC352, SNUG30017, XB6B4 |
| Stage 0 sporulation protein A | Shell (15) | 2789STDY5834960, AF31-21AC, AF36-10AT, AM22-21LB, AM37-1AC, AM43-11, BIOML-A1, BSD2780061698, J1101437, L1-82, M50_1, PC335, PC352, SNUG30017, XB6B4 |
| Chromosome-partitioning protein Spo0J | Shell (14) | 2789STDY5834960, AF31-21AC, AF36-10AT, AM22-21LB, AM37-1AC, AM43-11, BIOML-A1, J1101437, L1-82, M50_1, MSK_17_84, PC352, SNUG30017, XB6B4 |
| Sporulation transcription regulator WhiA | Shell (13) | 2789STDY5834960, AF31-21AC, AF36-10AT, AM22-21LB, AM37-1AC, BIOML-A1, BSD2780061698, J1101437, L1-82, M50_1, MSK_17_84, PC352, SNUG30017 |
| Spore protein YabP | Shell (13) | 2789STDY5834960, AF36-10AT, AM22-21LB, AM43-11, BIOML-A1, BSD2780061698, J1101437, L1-82, M50_1, MSK_17_84, PC335, PC352, XB6B4 |
| Stage V sporulation protein D | Shell (13) | 2789STDY5834960, AF31-21AC, AM37-1AC, AM43-11, BSD2780061698, J1101437, L1-82, M50_1, MSK_17_84, PC335, PC352, SNUG30017, XB6B4 |
| Cell division protein SepF | Shell (12) | 2789STDY5834960, AF36-10AT, BIOML-A1, BSD2780061698, J1101437, L1-82, M50_1, MSK_17_84, PC335, SNUG30017, XB6B4 |
| Spore protein YkvP | Shell (12) | 2789STDY5834960, AF31-21AC, AF36-10AT, AM22-21LB, AM43-11, BIOML-A1, BSD2780061698, L1-82, MSK_17_84, PC352, SNUG30017, XB6B4 |
| Sporulation initiation inhibitor protein Soj | Shell (12) | AF31-21AC, AF36-10AT, AM22-21LB, AM37-1AC, BIOML-A1, BSD2780061698, L1-82, M50_1, MSK_17_84, PC335, PC352, XB6B4 |
| Spore maturation protein A | Shell (12) | AF31-21AC, AM37-1AC, AM43-11, BSD2780061698, J1101437, L1-82, M50_1, MSK_17_84, PC335, PC352, SNUG30017, XB6B4 |
| Spore photoproduct lyase | Shell (12) | AF31-21AC, AF36-10AT, AM22-21LB, AM37-1AC, AM43-11, BIOML-A1, J1101437, L1-82, MSK_17_84, PC352, SNUG30017, XB6B4 |
| Sporulation initiation inhibitor protein Soj | Shell (11) | 2789STDY5834960, AF36-10AT, BIOML-A1, BSD2780061698, J1101437, L1-82, M50_1, MSK_17_84, PC335, PC352, XB6B4 |
| Sporulation initiation inhibitor protein Soj | Shell (10) | 2789STDY5834960, AF36-10AT, AM22-21LB, BIOML-A1, L1-82, M50_1, MSK_17_84, PC335, PC352, SNUG30017 |
| putative chromosome-partitioning protein ParB | Shell (10) | 2789STDY5834960, AF36-10AT, AM22-21LB, BIOML-A1, L1-82, M50_1, MSK_17_84, PC335, PC352, SNUG30017 |
| Sporulation-specific N-acetylmuramoyl-L-alanine amidase | Shell (10) | 2789STDY5834960, AF31-21AC, AF36-10AT, AM22-21LB, AM37-1AC, BIOML-A1, BSD2780061698, L1-82, PC352, XB6B4 |
| Chromosome-partitioning protein Spo0J | Shell (10) | AF36-10AT, AM37-1AC, BIOML-A1, BSD2780061698, J1101437, L1-82, M50_1, MSK_17_84, PC352, XB6B4 |

**Supplementary Table S7.** Flagella genes detected in the *R. intestinalis* pangenome.

| **Flagella Gene Description** | **Pangenome Set** | **Genomes Present In** |
| --- | --- | --- |
| Flagellar biosynthetic protein FliU | Core (16) | All |
| Flagellar basal body rod protein FlgB | Core (16) | All |
| Flagellar basal-body rod protein FlgC | Core (16) | All |
| flagellar hook-basal body protein FliE | Core (16) | All |
| flagellar MS-ring protein | Core (16) | All |
| Flagellar motor switch protein FliG | Core (16) | All |
| flagellar biosynthesis chaperone | Core (16) | All |
| Flagellar hook-length control protein FliK | Core (16) | All |
| flagellar basal body rod modification protein | Core (16) | All |
| Flagellar protein (FlbD) | Core (16) | All |
| flagellar basal body-associated protein FliL | Core (16) | All |
| Flagellar motor switch protein FliM | Core (16) | All |
| Flagellar motor switch protein FliN | Core (16) | All |
| Flagellar biosynthesis protein, FliO | Core (16) | All |
| Flagellar biosynthetic protein FliQ | Core (16) | All |
| flagellar biosynthesis protein FliR | Core (16) | All |
| Flagellar biosynthetic protein FlhB | Core (16) | All |
| Flagellar biosynthesis protein FlhA | Core (16) | All |
| Flagellar protein YcgR | Core (16) | All |
| Flagellar biosynthetic protein FliU | Core (16) | All |
| Flagellar biosynthetic protein FlhB | Core (16) | All |
| Flagellar hook-length control protein FliK | Core (16) | All |
| flagellar capping protein | Core (16) | All |
| flagellar protein FlaG | Core (16) | All |
| Flagellar assembly factor FliW | Core (16) | All |
| Flagellar hook-associated protein 3 | Core (16) | All |
| Flagellar hook-associated protein 1 | Core (16) | All |
| Flagellar hook-associated protein 1 | Core (16) | All |
| flagellar protein FliS | Core (16) | All |
| Flagellin N-methylase | Core (16) | All |
| Flagellar basal-body rod protein FlgG | Core (16) | All |
| Flagellar basal-body rod protein FlgG | Core (16) | All |
| Flagellin | Shell (10) | 2789STDY5834960, AF31-21AC, AM37-1AC, AM43-11, BSD2780061698, J1101437, MSK_17_84, PC335, PC352, SNUG30017 |
| Flagellin | Shell (10) | 2789STDY5834960, AF31-21AC, AM37-1AC, AM43-11, BSD2780061698, J1101437, MSK_17_84, PC335, PC352, SNUG30017 |
| Flagellar hook protein FlgE | Shell (11) | 2789STDY5834960, AF31-21AC, AM22-21LB, AM43-11, BSD2780061698, L1-82, M50_1, MSK_17_84, PC352, SNUG30017, XB6B4 |
| Flagellar protein FliS | Shell (14) | 2789STDY5834960, AF31-21AC, AM22-21LB, AM37-1AC, AM43-11, BSD2780061698, J1101437, L1-82, M50_1, MSK_17_84, PC335, PC352, SNUG30017, XB6B4 |
| Flagellar biosynthetic protein FliP precursor | Shell (15) | 2789STDY5834960, AF36-10AT, AM22-21LB, AM37-1AC, AM43-11, BIOML-A1, BSD2780061698, J1101437, L1-82, M50_1, MSK_17_84, PC335, PC352, SNUG30017, XB6B4 |
| Flagellar biosynthesis protein FlhF | Shell (15) | 2789STDY5834960, AF36-10AT, AM22-21LB, AM37-1AC, AM43-11, BIOML-A1, BSD2780061698, J1101437, L1-82, M50_1, MSK_17_84, PC335, PC352, SNUG30017, XB6B4 |
| Flagellum site-determining protein YlxH | Shell (15) | 2789STDY5834960, AF36-10AT, AM22-21LB, AM37-1AC, AM43-11, BIOML-A1, BSD2780061698, J1101437, L1-82, M50_1, MSK_17_84, PC335, PC352, SNUG30017, XB6B4 |
| flagellar protein FliS | Shell (15) | 2789STDY5834960, AF31-21AC, AF36-10AT, AM22-21LB, AM37-1AC, BIOML-A1, BSD2780061698, J1101437, L1-82, M50_1, MSK_17_84, PC335, PC352, SNUG30017, XB6B4 |
| Flagellin | Shell (15) | 2789STDY5834960, AF31-21AC, AF36-10AT, AM22-21LB, AM37-1AC, BIOML-A1, BSD2780061698, J1101437, L1-82, M50_1, MSK_17_84, PC335, PC352, SNUG30017, XB6B4 |
| Flagellin | Shell (3) | 2789STDY5834960, AF31-21AC, PC335 |
| Flagellin | Shell (3) | 2789STDY5834960, AF31-21AC, PC335 |
| Flagellin | Shell (3) | AM43-11, PC352, SNUG30017 |
| flagellar capping protein | Shell (3) | M50_1, MSK_17_84, PC352 |
| Flagellin | Shell (3) | 2789STDY5834960, AF31-21AC, PC335 |
| Flagellin | Shell (3) | AM43-11, PC352, SNUG30017 |
| Flagellar hook protein FlgE | Shell (4) | AF36-10AT, AM37-1AC, BIOML-A1, PC335 |
| Flagellar biosynthetic protein FliU | Shell (4) | 2789STDY5834960, AF31-21AC, AM37-1AC, PC352 |
| Flagellin | Shell (5) | AF36-10AT, BIOML-A1, L1-82, M50_1, XB6B4 |
| flagellar capping protein | Shell (5) | AM37-1AC, AM43-11, J1101437, L1-82, PC335 |
| Flagellin | Shell (5) | AF36-10AT, BIOML-A1, L1-82, M50_1, XB6B4 |
| flagellar capping protein | Shell (6) | 2789STDY5834960, AF31-21AC, AM22-21LB, BSD2780061698, SNUG30017, XB6B4 |
| Flagellar biosynthetic protein FliU | Shell (9) | AF36-10AT, AM43-11, BIOML-A1, BSD2780061698, J1101437, L1-82, M50_1, PC335, XB6B4 |
| Flagellin N-methylase | Shell (9) | 2789STDY5834960, AF31-21AC, AF36-10AT, AM22-21LB, AM43-11, BIOML-A1, J1101437, MSK_17_84, PC352 |

**Supplementary Table S8.** Chemotaxis genes detected in the *R. intestinalis* pangenome.

| **Chemotaxis Gene Description** | **Pangenome Set** | **Genomes Present In** |
| --- | --- | --- |
| Methyl-accepting chemotaxis protein 4 | Core (16) | All |
| Methyl-accepting chemotaxis protein 2 | Core (16) | All |
| Methyl-accepting chemotaxis protein PctB | Core (16) | All |
| Putative methyl-accepting chemotaxis protein YoaH | Core (16) | All |
| Putative methyl-accepting chemotaxis protein YoaH | Core (16) | All |
| Methyl-accepting chemotaxis protein III | Core (16) | All |
| Methyl-accepting chemotaxis protein III | Core (16) | All |
| Methyl-accepting chemotaxis protein 4 | Core (16) | All |
| Methyl-accepting chemotaxis protein 4 | Core (16) | All |
| Methyl-accepting chemotaxis protein IV | Core (16) | All |
| Methyl-accepting chemotaxis protein 4 | Core (16) | All |
| Methyl-accepting chemotaxis protein 4 | Core (16) | All |
| Methyl-accepting chemotaxis protein McpC | Core (16) | All |
| Methyl-accepting chemotaxis protein McpC | Core (16) | All |
| Methyl-accepting chemotaxis protein 4 | Core (16) | All |
| Methyl-accepting chemotaxis protein 3 | Core (16) | All |
| Methyl-accepting chemotaxis protein 4 | Core (16) | All |
| Methyl-accepting chemotaxis protein 4 | Core (16) | All |
| Chemotaxis protein CheV | Core (16) | All |
| Methyl-accepting chemotaxis protein PctB | Core (16) | All |
| Methyl-accepting chemotaxis protein II | Core (16) | All |
| Chemotaxis protein CheY | Core (16) | All |
| Methyl-accepting chemotaxis protein 4 | Core (16) | All |
| Methyl-accepting chemotaxis protein 1 | Core (16) | All |
| Chemotaxis protein methyltransferase Cher2 | Core (16) | All |
| Methyl-accepting chemotaxis protein IV | Core (16) | All |
| Methyl-accepting chemotaxis protein IV | Core (16) | All |
| Chemotaxis protein CheW | Core (16) | All |
| Chemoreceptor glutamine deamidase CheD | Core (16) | All |
| Chemotaxis protein CheW | Core (16) | All |
| Chemotaxis protein CheA | Core (16) | All |
| Chemotaxis response regulator protein-glutamate methylesterase | Core (16) | All |
| Chemotaxis protein CheY | Core (16) | All |
| Chemotaxis protein PomA | Core (16) | All |
| Putative methyl-accepting chemotaxis protein YoaH | Core (16) | All |
| Methyl-accepting chemotaxis protein IV | Core (16) | All |
| Methyl-accepting chemotaxis protein 4 | Core (16) | All |
| Methyl-accepting chemotaxis protein 4 | Core (16) | All |
| Methyl-accepting chemotaxis protein PctA | Core (16) | All |
| Methyl-accepting chemotaxis protein McpC | Core (16) | All |
| Methyl-accepting chemotaxis protein PctB | Shell (5) | AF36-10AT, AM37-1AC, PC335, SNUG30017, XB6B4 |
| Methyl-accepting chemotaxis protein PctB | Shell (4) | 2789STDY5834960, BIOML-A1, BSD2780061698, XB6B4 |
| Methyl-accepting chemotaxis protein IV | Shell (3) | AM22-21LB, AM43-11, J1101437 |
| Methyl-accepting chemotaxis protein IV | Shell (15) | 2789STDY5834960, AF31-21AC, AF36-10AT, AM22-21LB, AM37-1AC, AM43-11, BIOML-A1, BSD2780061698, J1101437, M50_1, MSK_17_84, PC335, PC352, SNUG30017, XB6B4 |
| Methyl-accepting chemotaxis protein IV | Shell (14) | 2789STDY5834960, AF31-21AC, AF36-10AT, AM37-1AC, AM43-11, BIOML-A1, BSD2780061698, L1-82, M50_1, MSK_17_84, PC335, PC352, SNUG30017, XB6B4 |
